# Phase 1 Safety and Immunogenicity Evaluation of ADMVA, a Multigenic, Modified Vaccinia Ankara-HIV-1 B'/C Candidate Vaccine

**DOI:** 10.1371/journal.pone.0008816

**Published:** 2010-01-25

**Authors:** Sandhya Vasan, Sarah J. Schlesinger, Zhiwei Chen, Arlene Hurley, Angela Lombardo, Soe Than, Phumla Adesanya, Catherine Bunce, Mark Boaz, Rosanne Boyle, Eddy Sayeed, Lorna Clark, Daniel Dugin, Mar Boente-Carrera, Claudia Schmidt, Qing Fang, Lei, Yaoxing Huang, Gerasimos J. Zaharatos, David F. Gardiner, Marina Caskey, Laura Seamons, Martin Ho, Len Dally, Carol Smith, Josephine Cox, Dilbinder Gill, Jill Gilmour, Michael C. Keefer, Patricia Fast, David D. Ho

**Affiliations:** 1 Aaron Diamond AIDS Research Center, New York, New York, United States of America; 2 The Rockefeller University, New York, New York, United States of America; 3 International AIDS Vaccine Initiative, New York, New York, United States of America; 4 University of Rochester Medical Center, Rochester, New York, United States of America; 5 Weill Cornell Medical Center, New York, New York, United States of America; 6 International AIDS Vaccine Initiative Core Laboratory, Imperial College, London, United Kingdom; 7 EMMES Corporation, Rockville, Maryland, United States of America; University of New South Wales, Australia

## Abstract

**Background:**

We conducted a Phase I dose-escalation trial of ADMVA, a Clade-B'/C-based HIV-1 candidate vaccine expressing *env*, *gag*, *pol*, *nef*, and *tat* in a modified vaccinia Ankara viral vector. Sequences were derived from a prevalent circulating HIV-1 recombinant form in Yunnan, China, an area of high HIV incidence. The objective was to evaluate the safety and immunogenicity of ADMVA in human volunteers.

**Methodology/Principal Findings:**

ADMVA or placebo was administered intramuscularly at months 0, 1 and 6 to 50 healthy adult volunteers not at high risk for HIV-1. In each dosage group [1×10^7^ (low), 5×10^7^ (mid), or 2.5×10^8^ pfu (high)] volunteers were randomized in a 3∶1 ratio to receive ADMVA or placebo in a double-blinded design. Subjects were followed for local and systemic reactogenicity, adverse events including cardiac adverse events, and clinical laboratory parameters. Study follow up was 18 months. Humoral immunogenicity was evaluated by anti-gp120 binding ELISA, immunoflourescent staining, and HIV-1 neutralization. Cellular immunogenicity was assessed by a validated IFNγ ELISpot assay and intracellular cytokine staining. Anti-vaccinia binding titers were measured by ELISA.

ADMVA was generally well-tolerated, with no vaccine-related serious adverse events or cardiac adverse events. Local or systemic reactogenicity events were reported by 77% and 78% of volunteers, respectively. The majority of events were of mild intensity. The IFNγ ELISpot response rate to any HIV antigen was 0/12 (0%) in the placebo group, 3/12 (25%) in the low dosage group, 6/12 (50%) in the mid dosage group, and 8/13 (62%) in the high dosage group. Responses were often multigenic and occasionally persisted up to one year post vaccination. Antibodies to gp120 were detected in 0/12 (0%), 8/13 (62%), 6/12 (50%) and 10/13 (77%) in the placebo, low, mid, and high dosage groups, respectively. Antibodies persisted up to 12 months after vaccination, with a trend toward agreement with the ability to neutralize HIV-1 SF162 *in vitro*. Two volunteers mounted antibodies that were able to neutralize clade-matched viruses.

**Conclusions/Significance:**

ADMVA was well-tolerated and elicited durable humoral and cellular immune responses.

**Trial Registration:**

Clinicaltrials.gov NCT00252148

## Introduction

With an estimated 33 million people living with HIV/AIDS globally, and approximately 2.5 million new infections in 2007 alone, the need for an effective vaccine to prevent or attenuate HIV-1 infection remains paramount [1]. In the People's Republic of China, an estimated 700,000 people are living with HIV/AIDS in an epidemic spread both through sexual transmission and injection drug use. The prevalence of HIV infection among injection drug users in Yunnan province, which borders Myanmar, Laos, and Vietnam in the “golden triangle” region, has increased dramatically in the last ten years, to over 40% in several prefectures [2]. In a separate study, the incidence rate of new HIV infections among intravenous drug users in Guanxgi province was found to be 3.1% [3].

For these reasons, our laboratory has pursued the development of a multigenic vaccine regimen based on the predominant B'/C circulating recombinant form of HIV-1 from Yunnan, China, CRF07_BC [4]. After codon-optimization and certain safety mutations, matched sequences from the *env*, *gag*, *pol*, *nef*, and *tat* genes were inserted into both a naked DNA plasmid backbone (ADVAX) and a modified vaccinia ankara (MVA) viral vector (ADMVA), as described by Y. Huang et al. and Z. Chen et al., respectively [5], [6]. These vectors were initially chosen based on reports of improved cellular immunogenicity when used in a prime-boost combination in humans with a variety of antigens [7]–[9] and on their ability to control viremia after multiple routes of SHIV challenge in rhesus macaques [10], [11].

The Phase I trial described in this report was designed to assess the safety, tolerability, and humoral and cellular immunogenicty of ADMVA alone. A parallel Phase I study of the ADVAX vaccine alone was conducted separately, as reported in the accompanying manuscript.

## Methods

### Study Setting

The study was conducted at the Rockefeller University Hospital in New York City, USA, and at the University of Rochester Medical Center in Rochester, New York, USA. The protocol for this trial and supporting CONSORT checklist are available as supporting information; see Checklist S1 and Protocol S1. This trial is registered at clinicaltrials.gov, registry number NCT00252148, http://clinicaltrials.gov/ct2/show/NCT00252148.

### Participants

Healthy men and women aged 18–40 years were eligible for participation if they were not at high risk for HIV-1, as defined by having none of the following activities in the six months prior to enrollment: unprotected vaginal or anal sex with a known HIV-1-infected person or casual partner, injection drug use, acquisition of a sexually transmitted disease, or sex work for money or drugs. Participants agreed to safe sexual practices and to use effective contraception to avoid pregnancy throughout the duration of the 18-month study. Participants had to demonstrate a clear understanding of the possibility of HIV-1 seroconversion in the event of a humoral immune response to encoded HIV-1 antigens. Exclusion criteria included chronic medical conditions, clinically significant abnormal laboratory parameters, infection with Hepatitis B or C, infection with syphilis, or recent receipt of a vaccine or blood transfusion. Although MVA has not been associated with myocarditis or pericarditis to date, due to the rare occurrence of cardiac events after vaccination with live replicating vaccinia to prevent smallpox infection [12], [13], volunteers with abnormal electrocardiograms, troponin values, or a history of cardiac abnormalities were also excluded from this study. Individuals with a prior history of smallpox immunization were limited to no more than ten percent of all volunteers.

### Ethical Compliance

This study was approved by the Institutional Review Boards of the Rockefeller University Hospital and the University of Rochester Medical Center. All participants in this study provided written informed consent after appropriate review, discussion and counseling by the clinical study team. The trial was monitored by the International AIDS Vaccine Initiative (IAVI) and conducted in compliance with International Conference on Harmonisation - Good Clinical Practice (ICH-GCP).

### Interventions

The ADMVA vaccine is a non-replicating viral vaccine constructed with the MVA backbone expressing sequences from the *env*, *gag*, *pol*, *nef*, and *tat* genes of HIV-1 B'/C, as previously described [6]. GMP manufacturing, quality control testing and real-time stability studies of ADMVA clinical lots were undertaken at Impfstoffwerk Dessau-Tornau GmbH (IDT-Germany).

The study was randomized, dose-escalating, and double-blinded with respect to active vaccine or placebo. Study site staff and volunteers remained blinded with respect to the allocation of placebo or vaccine, but not dosage group. Safety and tolerability of ADMVA or placebo in each dosage group were evaluated by an independent Data and Safety Monitoring Board at least 14 days after the12th volunteer had received the second injection, and prior to initiation of enrollment of the next dose group. The study design is summarized in Table 1.

**Table 1:** Study design.

Group	Vaccine Dose (pfu)	Volunteers Receiving Vaccine:Placebo	Vaccination Schedule(Months)	Total Follow Up (Months)
Low	1.0×10^7^	12∶4	0, 1, 6	18
Middle	5.0×10^7^	12∶4	0, 1, 6	18
High	2.5×10^8^	12∶4	0, 1, 6	18
**Total**		36∶12		

Note: An over enrollment of 10% was allowed to compensate for discontinuation of vaccinations within 30 days of enrollment.

### Objectives

The primary objective was to evaluate the safety and tolerability of three vaccinations with ADMVA at three different dosage levels in healthy HIV-uninfected adults. The secondary objective was to evaluate the humoral and cellular immunogenicity of ADMVA versus placebo at each dose.

### Outcomes

Primary endpoints were designed to evaluate the safety of ADMVA in human volunteers. Local reactogenicity (including pain, tenderness, erythema, edema, skin damage, induration, and formation of crust, scab or scar) and systemic reactogenicity (including fever, chills, headache, nausea, vomiting, malaise, fatigue, myalgia, arthralgia, rash, chest pain, palpitations, reduced exercise, shortness of breath and allergic reaction) were assessed by telephone two to four days following each vaccination and by history and physical examination one and two weeks after each vaccination. Subjects were monitored for adverse events, general health and laboratory parameters at each study visit. Due to reports of myo- and pericarditis following vaccination with live replicating vaccinia virus [12], [13], subjects were also monitored for evidence of cardiac abnormalities.

Secondary endpoints were designed to evaluate the cellular and humoral immunogenicity of ADMVA. Cellular immunogenicity was assessed by IFNγ ELISpot on frozen peripheral blood mononuclear cells (PBMCs) at the IAVI Core Laboratory at the Imperial College, London, as previously described [14], and as detailed in the accompanying manuscript in this issue.

#### Cell stimulation

ELISpot-positive samples were tested for phenotype, cytokine secretion, and antigen-specific proliferation using polychromatic flow cytometry as described in the accompanying manuscript in this issue.

#### Humoral immunogenicity

Antibodies to Clade C gp120 (NIH AIDS Reagent Program) were assessed by ELISA at pre-vaccination baseline and two weeks after each vaccination, as described by Huang et al. [15]. In parallel, anti-gp160, anti-p24, or anti-gp36 Group M/O antibodies were assessed using the Genetic Systems™ HIV-1| HIV-2 PLUS O EIA Kit (Bio-Rad Laboratories, Hercules, CA), at the New York State Department of Health. Those samples that were positive were further evaluated by the Genetic Systems™ HIV-1 Western Blot Kit (Bio-Rad Laboratories, Hercules, CA) and for viral load quantification using the Roche Amplicor HIV-1 Monitor v1.5 RNA-PCR Kit (Roche Diagnostic Systems, Indianapolis, IN) to differentiate a response to vaccine from *de novo* HIV infection. Results were monitored by an independent physician to maintain blinding of the clinical study team.

Serum from pre-vaccination and from four weeks after the third vaccination was assessed for neutralization of a panel of laboratory strain and primary HIV-1 Clade C and Clade B isolates at Monogram Biosciences, Inc. (San Francisco, CA) [16]. Development of anti-vaccinia binding antibodies was quantified by a binding antibody ELISA performed by V-Bio, Inc. (St. Louis, MO).

Antibodies against conformational envelope were detected by an immunoflourescent staining assay for Vero cells expressing envelope. Vero cells were transfected with a DNA plasmid expressing Clade C/B' envelope. After 48 hours, cells were fixed and incubated with undiluted serum for 37°C for one hour. Antibodies bound to envelope were detected by an anti-human IgG fluorescent dye (Alexa Fluor 594 goat anti-human IgG, Invitrogen, Carlsbad, CA).

### Sample Size

In each of the three dosage groups volunteers were randomized in a 3∶1 ratio of active vaccine to placebo. The study design allowed for a total of 48 volunteers to be enrolled; 36 volunteers receiving active vaccines and 12 volunteers receiving placebo. However, up to ten percent over enrollment was permitted to compensate for discontinuation of vaccinations within 30 days of enrollment, resulting in an extra vaccine recipient in the low and high dosage groups, making the total sample size 50. The small sample size was deemed adequate for an exploratory dose-escalation study of a novel product while investigating safety and tolerability of the vaccine. Based on a 10% event rate in the placebo group (n = 12), there was at least 80% power to detect a significantly greater event rate of 51% or more in the active group (n = 36) at level α = 0.05 using Fisher's exact one-sided test.

### Randomization and Blinding

The randomization schedule was prepared by the statisticians at the Data Coordinating Center at the EMMES Corporation. The randomization list was sent to Fisher Clinical Services, Inc. for labeling and packaging of study vaccine and placebo in a double-blind fashion. Study site staff, volunteers, and laboratories remained blinded with respect to the allocation of placebo or vaccine, but not dosage group.

### Statistical Methods

Data from all participants, including those lost to follow up and those not completing the vaccination series, were included in the analyses. The distribution of overall maximum severity per volunteer of local and systemic reactogenicity events was used to assess the differences between dosage groups. Fisher's exact test was used for 2×2 tables, and the Cochran-Armitage trend test was used to investigate trends in event rates with increasing dosage. The Kappa statistic and McNemar's test were used for tests of agreement.

## Results

### Participant Flow

**Figure 1:**
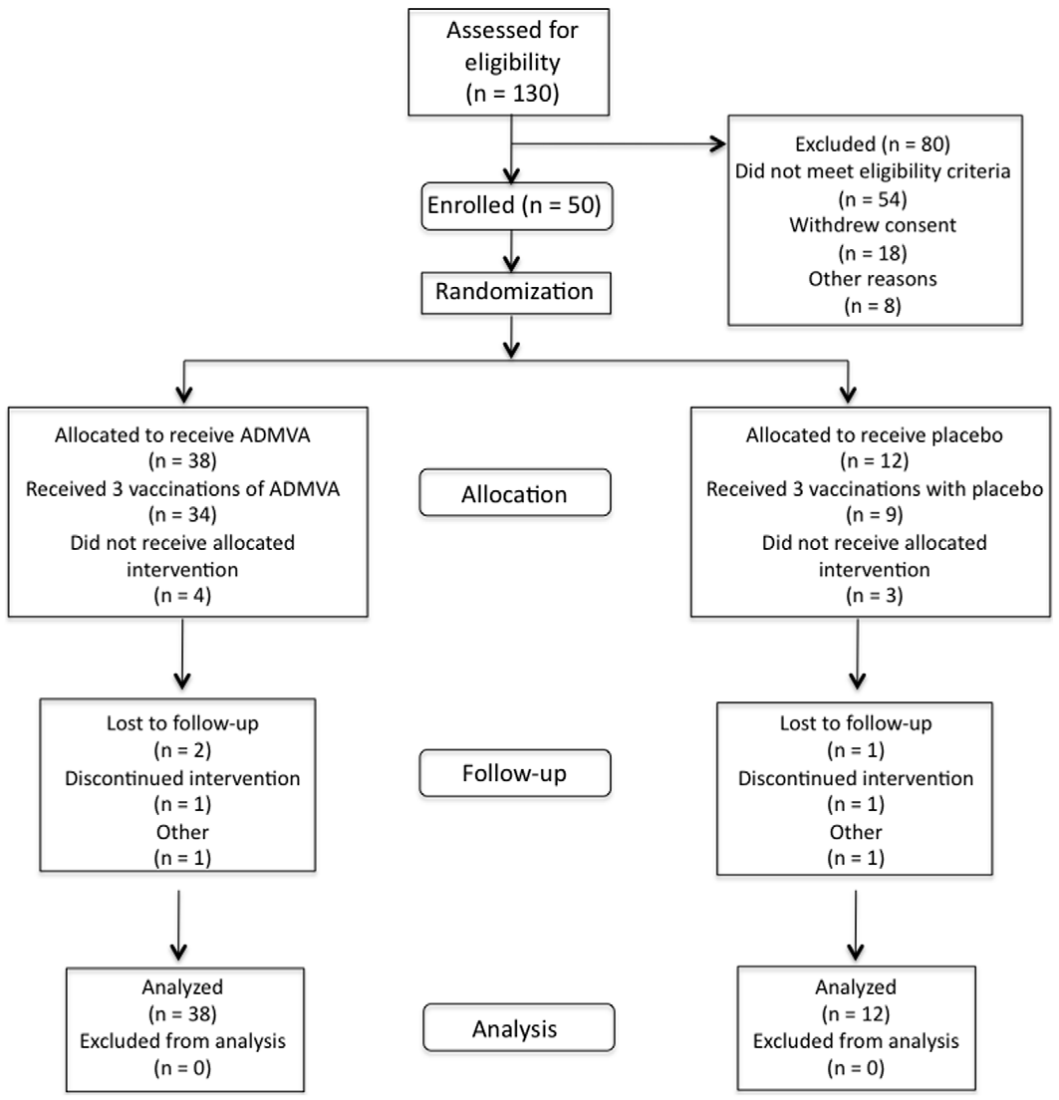
Clinical trial participant flow diagram.

As shown in Figure 1, 130 volunteers were screened for this study, of whom 50 volunteers were enrolled. The majority of the 80 screen failures were due to medical abnormalities: 19 due to chronic medical conditions, 24 due to abnormalities on screening laboratories or urinalysis, and 11 due to minor abnormalities on ECG. Eighteen volunteers withdrew consent after completing the screening process. Of the remaining eight screen failures, seven were assessed by the study team as being unable to comply with the protocol, and one was already enrolled in another clinical trial of an investigational agent. The average interval from date of screening to enrollment was 18 days, ranging from 6–42 days. All 13 low dosage volunteers completed the three planned vaccinations. In the mid dosage group, 2 volunteers received only two and one received only one vaccination. In the high dosage group, one volunteer received only 2 vaccinations. One placebo recipient withdrew after the first vaccination due to a non-related serious adverse event. Another placebo recipient missed the second vaccination, but received the third. None of the discontinuations was related to study vaccine.

### Recruitment

Enrollment started in January 2005 and was completed in January 2006. Study follow up ended in August 2007. Baseline demographic and clinical characteristics for all trial participants are listed in Table 2.

**Table 2:** Subject demographics.

	ADMVA Low	ADMVA Mid	ADMVA High	Placebo	All Subjects
**Gender**					
Male	6	6	8	6	26
Female	7	6	5	6	24
**Age**					
Mean	27.6	24.8	25.1	25.8	25.8
Range	19–40	19–40	21–32	18–40	18–40
**Race/Ethnicity**					
Caucasian	6	2	6	6	20
Asian	1	0	2	1	4
African American	1	6	2	2	11
Hispanic or Latino	3	4	2	2	11
Native American or Alaskan Native	1	0	0	0	1
Native Hawaiian or Other Pacific Islander	1	0	0	0	1
Other/Unknown	0	0	1	1	2

### Reactogenicity and Adverse Events

ADMVA was generally well tolerated at all dosages. Two volunteers, both randomized to receive placebo, experienced serious adverse events not related to vaccination (pituitary tumor and brain tumor, both likely undiagnosed pre-existing conditions). The remainder of adverse events were mild (132/176 events, 75%) and not related or unlikely related to vaccine (165/176 events, 94%). There was no clinical or laboratory evidence of pericarditis or myocarditis.

**Figure 2:**
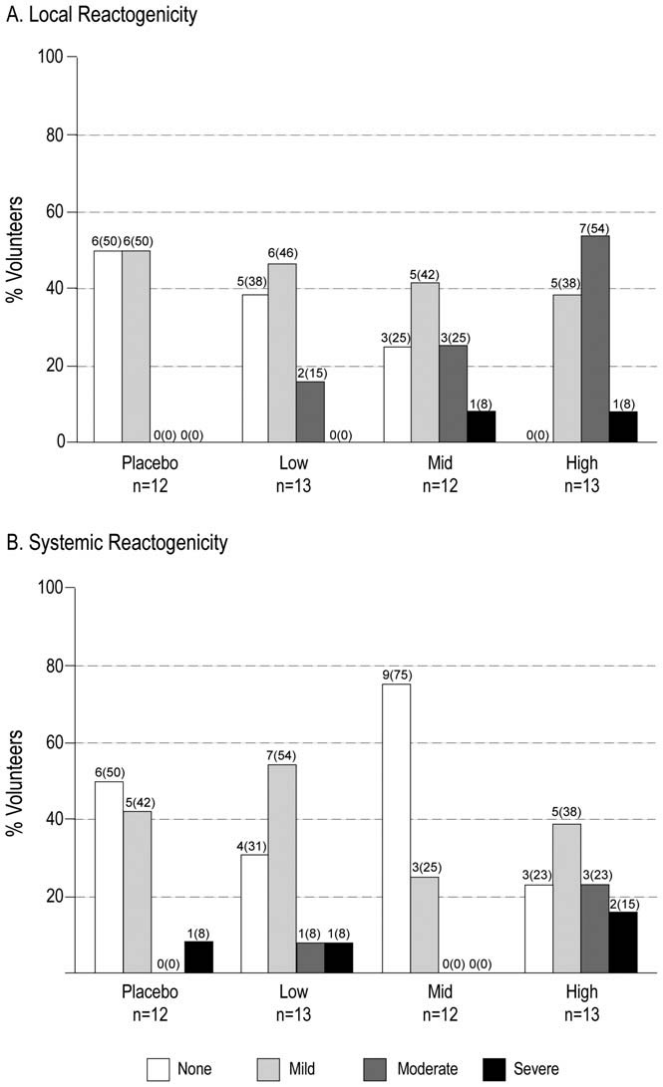
Local and systemic reactogenicity by dosage group. Panels A and B depict the percentage of volunteers experiencing local or systemic reactogenicity, respectively, by severity and dosage group. Total responses and (percentage of responses) are depicted above each bar. There is evidence of increased moderate/severe local reactions with increasing dose (two-tailed Cochran-Armitage trend test: p = 0.015). A similar comparison of systemic reactogenicity was not statistically significant (p = 0.129).

The percentage of volunteers experiencing local and systemic reactogenicity after each vaccine is presented in Figure 2. The most frequently reported local reactogenicity events in all dosage groups were pain and tenderness. The most frequently reported systemic reactogenicity events in all dosage groups were headache, fever, myalgia and fatigue, all of which were generally mild. Local and systemic reactogenicity events generally resolved within 4 days after vaccination. The proportion of volunteers with moderate/severe local reactions increased significantly with increasing dosage (15%, 33% and 62% in the low, mid and high dose groups, respectively: p = 0.015), whereas dose had no significant effect on moderate/severe systemic reactogenicity (p = 0.129).

### Cellular Immunogenicity

IFNγ ELISpot results are summarized in Table 3. In the low dosage group, three of twelve vaccinees (25%) formed ELISpot responses to HIV envelope (mean 79, range 57–138 SFC/million). One volunteer in the low dose group was excluded from ELISpot analysis due to QC failure secondary to high background. Six of twelve vaccinees (50%) in the mid dosage group (mean 69, range 40–394 SFC/million) and eight of thirteen vaccinees (62%) in the high dosage group mounted IFNγ responses to multiple gene products (mean 89, range 42–275 SFC/million). There were no positive responses to any peptide pool among the placebo recipients. The majority of the responses in the low and mid dosage groups occurred after at least the second vaccination. In the high dosage group, IFNγ ELISpot responses in 5/8 responders occurred as early as 1–2 weeks after the first vaccination. Intracellular cytokine responses were undetectable in all ELISpot-positive volunteers.

**Table 3:** IFNγ ELISpot results.

ADMVA dosage groups (pfu)	1×10^7^	5×10^7^	2.5×10^8^
Positive volunteers	3/12 (25%)	6/12 (50%)	8/13 (62%)
SFC per million – mean	79	69	89
SFC per million – range	(57–138)	(40–394)	(42–275)
Gag responders	0	2	0
Env responders	3	4	6
Pol responders	0	3	3
Nef-Tat responders	0	1	3
Response Timing – median (week)	27	27	6
Response Timing – range (weeks)	6–28	2–78	1–78

**Table 3** summarizes the IFNγ ELISpot response rate and magnitude in spot forming cells per million PBMCs (SFC) among volunteers receiving ADMVA by dose group. There were no positive responses in placebo recipients. The timing of IFNγ ELISpot responses and distribution of antigens eliciting these responses are listed.

### Humoral Immunogenicity

#### Binding antibodies

As shown in Table 4, eight of thirteen volunteers (62%) in the low dosage group, six of twelve volunteers (50%) in the mid dosage group, and ten of thirteen (77%) in the high dosage group formed binding antibodies against HIV-1 subtype C gp120. None of the placebo recipients formed positive responses. Total response rates in the low, mid and high dosage groups in either the IFNγ ELISpot assay or the anti-gp120 binding assay were 10/13 (77%), 7/12 (58%), and 12/13 volunteers (92%), respectively. Anti-gp120 binding antibodies were elicited in all three dose groups after the three injections of ADMVA, although one responder formed antibodies after one vaccination with high dose ADMVA, and five responders formed anti-gp120 antibodies after two vaccinations. All responders formed antibodies to conformationally intact HIV-1 B'/C envelope expressed on Vero cells, as measured by immunofluorescent staining.

**Table 4:** Binding and neutralizing antibody response rate.

Vaccine Dose	Anti-gp120 Ab (%)	SF162 Neutralization (%)	HIV-1 Clade C Neutralization (%)
Placebo	0/12 (0%)	0/10 (0%)	0/12 (0%)
1.0×10^7^	8/13 (62%)	5/13 (39%)	1/12 (8%)
5.0×10^7^	6/12 (50%)	6/11 (55%)	1/12 (8%)
2.5×10^8^	10/13 (77%)	10/12 (83%)	0/13 (0%)

One ADMVA vaccine recipient in the high dosage group tested positive on a standard clinical HIV ELISA two weeks after the third vaccination. This Western Blot showed positive bands against gp120 and p24, but the viral load as measured by HIV-1 RT-PCR was undetectable. All subsequent ELISA results in this volunteer were negative. At the final study visit, no volunteers tested HIV positive.

#### Neutralizing antibodies

Table 4 also depicts the frequency of volunteers with neutralizing antibodies to HIV-1 laboratory strain SF162, and to a subtype C HIV-1 isolate. After three vaccinations of ADMVA, two volunteers were able to neutralize the subtype C viruses, and three volunteers (one placebo and two high dose volunteers) were able to neutralize HIV-1 laboratory strain NL43. 21/36 ADMVA recipients (58%) were able to neutralize the laboratory HIV strain SF162 at Week 28, which trended towards agreement with the formation of anti-gp120 binding antibodies (Kappa = 60%, McNemar's test p = 0.7). Reciprocal geometric mean neutralizing titers were all low (<300 except for one titer of 476) against the SF162 in the low, mid and high dose groups and <20 in all placebo specimens.

#### Anti-vaccinia antibodies

**Figure 3:**
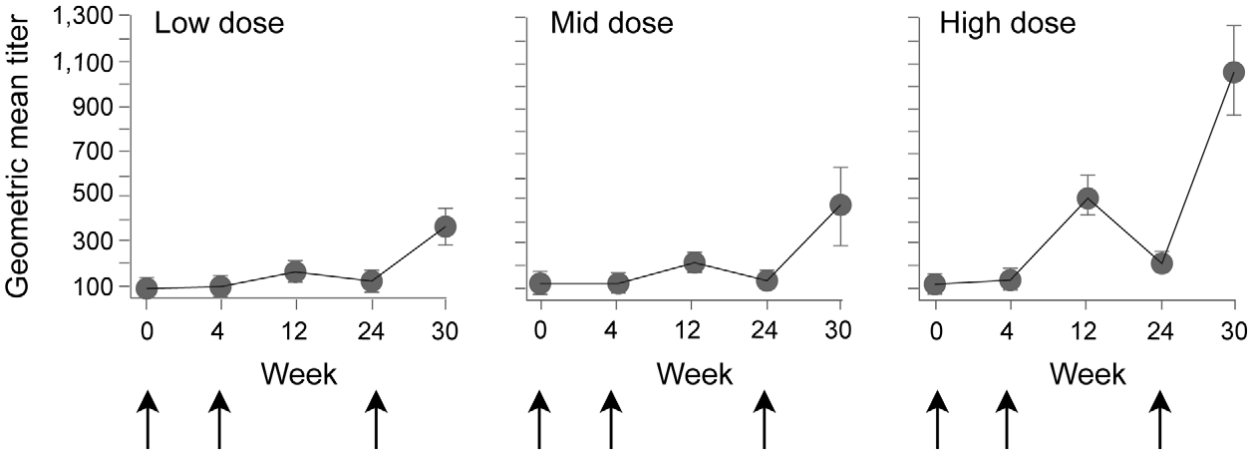
Graphs depict the anti-vaccinia binding antibody titer after each vaccination (arrows) by dose group, expressed as geometric mean titer. Error bars represent SEM. Arrows indicate vaccination time points. As predicted, anti-vaccinia antibody titers increased after each vaccination and with increasing doses of ADMVA.

Figure 3 depicts the average anti-vaccinia antibody titer over time in each dosage group. As expected, anti-vaccinia titers increased after each subsequent immunization in a dosage-dependent manner. In the placebo group, 1/12 (8.3%) was positive at baseline and throughout the trial. No other placebos were positive after immunization. In the low dose group, 3/12 (25%) were positive at baseline and 12/12 after immunization (100%). One volunteer in the low dose group was excluded from analysis due to unavailability of sample. In the mid dose group, 1/12 (8.3%) were positive at baseline and 11/12 (91.6%) after immunization. In the high dose group, none were positive at baseline and 13/13 (100%) were positive after immunization. Interestingly, there was no correlation between individuals with a prior history of smallpox vaccination and positive baseline anti-vaccinia titers.

## Discussion

This trial was the first evaluation of ADMVA in human volunteers. ADMVA was well tolerated at the dosage levels tested, with no evidence of cardiac toxicity. There were no serious adverse events related to vaccine. Local and systemic reactogenicity following vaccination was usually mild to moderate and generally resolved within four days. Local reactogenicity increased in severity with each dosage group. This dosage-dependent reactogenicity may indicate an immune response to products of the HIV gene inserts, to the viral vector, or both. While anti-vector immunity increased after subsequent vaccinations in each dose group, a titer of 1∶450 in the high dose group after two vaccinations did not prevent generation of humoral or cellular immune responses after the third vaccination.

In the mid and low dosage groups, binding antibodies to gp120 were detected only after the third dose of ADMVA, while in a subset of volunteers in the high dosage group, binding antibodies were detected after the first and/or second vaccinations, although the majority of vaccine recipients also required three injections. Antibody titer peaked two weeks after vaccination and then waned, but persisted for one year post vaccination. Binding antibodies were likely functional in part, given the correlation with the ability to neutralize HIV SF162, a strain that is relatively easy to neutralize. Given the inability to neutralize clade-matched HIV isolates in the majority of volunteers, it is unlikely that this humoral response will be sufficient on its own to neutralize incoming infection, reduce viral load set point, or impact disease progression post infection with HIV-1.

ADMVA elicits a cellular immune response, as quantified by IFNγ ELISpot assay. Responses occurred after one, two and three vaccinations, and were directed against multiple antigens. Unfortunately, in both humans and macaques, IFNγ ELISpot responses do not correlate with protection from HIV/SIV or reduction in viral load [17]–[19]. The magnitude of the ELISpot response may also not reflect the quality of the cellular immune response [20], [21]. In our hands, the 16-hour detection platform of the ELISpot is more sensitive for IFNγ detection than the 6-hour detection platform of the flow assay, which may account for the lack of detectable responses on intracellular cytokine staining. As there has been no documented case of natural clearance of HIV-1 in humans, much remains to be understood regarding the immunologic correlates of protection from HIV-1. Therefore, many of the current vaccine strategies to induce cellular immune responses are in effect, proceeding “blinded”, as we do not yet know the desired immune response.

Given the possibility of enhanced susceptibility to HIV infection in adenoviral vaccine recipients with high pre-existing adenoviral titers [18], [19], studies of HIV vaccines in humans should not be pursued without sufficient consideration for volunteer safety. Poxviral vectors have fewer issues with pre-existing immunity, as such immunity is generally limited to persons who have been previously vaccinated against smallpox. Since routine smallpox vaccinations have been discontinued for several decades worldwide, with the exception of certain groups perceived to be “at risk”, such as military personnel and health care workers [22], the prevalence of pre-existing immunity to an MVA-based vaccine would arguably be low, relative to adenovirus-based vaccines [23].

This vaccine was designed to be administered in combination with ADVAX, a matched Clade C-B' DNA-based multigenic vaccine (see the accompanying manuscript). Given that the DNA prime - MVA boost vaccinations have proven superior to MVA vaccinations alone in animal models and in humans [7]–[11], [24], [25], it is possible that ADMVA may be more immunogenic when administered in combination with other DNA or viral vectors.

## Supporting Information

Checklist S1 CONSORT Checklist.
(0.19 MB DOC)

Protocol S1 Trial Protocol.
(7.99 MB PDF)
