# Supplementary material for: Phase 1 Safety and Immunogenicity Evaluation of ADMVA, a Multigenic, Modified Vaccinia Ankara-HIV-1 B'/C Candidate Vaccine
Source: PLoS One. 2010 Jan 25;5(1):e8816. doi: 10.1371/journal.pone.0008816 (PMC2810329; doi:10.1371/journal.pone.0008816)
Supplement: Protocol S1 — Trial Protocol. (7.99 MB PDF) [file pone.0008816.s002.pdf]

MVA (ADMVA) Dose-Escalating Study

**Protocol Title:**	A Randomized, Placebo-Controlled, Dose-Escalating, Double-Blinded Phase 1 Study to Evaluate the Safety and Immunogenicity of a Modified Vaccinia Ankara (MVA) expressing HIV-1 Clade C env/gag-pol and nef-tat fusion genes (ADMVA) Vaccine Administered Intramuscularly to HIV-Uninfected, Healthy Volunteers.
**Protocol Number:**	IAVI C002
**Phase:**	Phase 1
**Sponsor:**	International AIDS Vaccine Initiative (IAVI)<br>110 William Street, 27th Floor<br>New York, New York<br>10038-3901<br>USA
**Date of Protocol Version 1.0:**	25 June 2004
**Date of Protocol Amendment Version 1.1:**	1 September 2004
**Date of Protocol Amendment Version 1.2:**	2 November 2004
**Date of Protocol Amendment Version 1.3:**	5 January 2005
**Date of Protocol Amendment Version 1.4:**	14 March 2005

UNCONTROLLED COPY

SYNOPSIS	
**TITLE:**	A Randomized, Placebo-Controlled, Dose-Escalating, Double-Blinded Phase 1 Study to Evaluate the Safety and Immunogenicity of a Modified Vaccinia Ankara (MVA) expressing HIV-1 Clade C env/gag-pol and nef-tat fusion genes (ADMVA) Vaccine Administered Intramuscularly to HIV-Uninfected, Healthy Volunteers.
**PROTOCOL NUMBER:**	IAVI C002
**PHASE:**	Phase 1
**INVESTIGATOR SITE:**	Aaron Diamond AIDS Research Center (ADARC)<br>The Rockefeller University Hospital<br>The Rockefeller University, 1230 York Avenue<br>New York, NY 10021<br><br>University of Rochester Medical Center<br>601 Elmwood Ave.<br>Rochester, NY 14642
**SPONSOR:**	International AIDS Vaccine Initiative (IAVI)<br>110 William Street, 27th Floor<br>New York, NY 10038-3901<br>USA
**OBJECTIVES:**	***Primary:***<br>To evaluate the safety of ADMVA administered three times, intramuscularly at 3 dosage levels.<br><br>**Secondary:**<br>To evaluate the immunogenicity of ADMVA administered three times, intramuscularly at 3 dosage levels.
**ENDPOINTS:**	***Primary:***<br>***Safety and tolerability:***<br>▪ The proportion of volunteers with severe local reactogenicity (e.g. pain/tenderness, erythema, edema, induration, skin lesion [vesiculation/ulceration], formation of crust, scab or scar).<br>▪ The proportion of volunteers with severe systemic reactogenicity

SYNOPSIS	
	(e.g. fever, chills, headache, nausea, vomiting, malaise, fatigue, myalgia, rash, arthralgia).<br>▪ The proportion of volunteers with severe adverse events (including laboratory abnormalities).<br>▪ The proportion of volunteers with serious adverse events.<br>▪ The proportion of volunteers with mild and moderate local and systemic reactogenicity events and adverse events.<br><br>***<u>Secondary:</u>***<br>***Immunogenicity:***<br>▪ The proportion of volunteers with HIV-1 specific T- cell responses quantified by ELISPOT.<br>▪ The proportion of volunteers with HIV-1 specific T-cell responses quantified by cytokine flow cytometry (CFC).<br>▪ The proportion of volunteers with binding and neutralizing antibody responses.<br>▪ All immune responses will be evaluated for proportion of responders and the mean responses will be compared.<br>Serum antibodies and T cell responses against the MVA vector may be examined using ELISA, viral neutralization assays, ELISPOTS and/or cytokine flow cytometry (CFC) at selected time points.
**STOPPING CRITERIA FOR STUDY:**	▪ If there is one SAE graded as severe and judged as possibly, probably, or definitely related to study vaccine by the principal investigator or designee, the trial will be suspended pending a review by at least one member of the DSMB which may be unblinded at its discretion. Following this review, the DSMB member(s) will make a recommendation to the principal investigators and IAVI regarding the continuation of the trial.

UNCONTROLLED COPY

SYNOPSIS

STUDY DESIGN TABLE:

Group	Vaccine/ Placebo	Month 0*	Month 1*	Month 6*
Low	12/4	$1X10^7$pfu ADMVA or Placebo IM	$1X10^7$pfu ADMVA or Placebo IM	$1X10^7$pfu ADMVA or Placebo IM
Middle	12/4	$5X10^7$pfu ADMVA or Placebo IM	$5X10^7$pfu ADMVA or Placebo IM	$5X10^7$pfu ADMVA or Placebo IM
High	12/4	$2.5X10^8$pfu ADMVA or Placebo IM	$2.5X10^8$pfu ADMVA or Placebo IM	$2.5X10^8$pfu ADMVA or Placebo IM
Total Number	36/12	*ADMVA or Placebo given IM at the dose indicated		

STUDY POPULATION:

Healthy male or female adults 18-40 years of age, who in the opinion of the principal investigator or designee, understand the study and can provide written informed consent. Principal exclusion criteria include: HIV-1 or HIV-2 infection; pregnancy and lactation; chronic disease; recent vaccination or receipt of a blood product or experimental agent; previous severe vaccine reaction and reported high risk behavior for HIV infection.

NUMBER OF VOLUNTEERS:

Approximately 48 volunteers (36 vaccine recipients/12 placebo recipients) will be enrolled in the study. An over-enrolment of up to 10% (5 additional volunteers) will be permitted in the study.

DESCRIPTION OF INVESTIGATIONAL PRODUCT:

ADMVA is a recombinant MVA vaccine expressing HIV-1 env/gag-pol and nef-tat fusion genes based on a Chinese HIV-1 Clade B'/C isolate, CRF 007.

The Placebo is 10mM Tris HCl, 140mM NaCl, pH 7.7.

Vaccine/ Placebo	Dosage Level	Total Volume in Vial (ml)	Total Injected Volume (ml)	Route of Administration
ADMVA	$1x10^7$ pfu	0.7ml	0.5ml	IM
	$5x10^7$ pfu	0.7ml	0.5ml	IM
	$2.5x10^8$ pfu	0.7ml	0.5ml	IM
Placebo	NA	0.7ml	0.5ml	IM

IM= Intramuscular

SYNOPSIS	
**DOSE ESCALATION:**	16 volunteers will be enrolled in each of up to three dose groups and randomized in a 3:1 ratio of active vaccine to placebo. Safety and tolerability of the ADMVA vaccine/placebo will be evaluated at least 14 days after the 12th volunteer in the low dose group receives the second injection before proceeding to the middle dose group. Likewise, there will be an evaluation at least 14 days after the 12th volunteer in the middle dose group receives the second injection before proceeding to the high dose group.
**BLINDING:**	This is a dose escalation trial. Study site staff and volunteers will be blinded only with respect to the allocation of placebo or vaccine. Binding will not apply to the assignment of dose levels (low, middle or high).
**DURATION OF STUDY PARTICIPATION:**	Volunteers will be screened up to 42 days before enrolment and will be followed for 18 months after the first vaccination.
**EVALUATION FOR INTERCURRENT HIV INFECTION:**	Volunteers will be tested for HIV-1 and HIV-2 antibodies according to the Schedule of Procedures (Appendix A) or if medical or social circumstances dictate, using two different ELISA tests. Should one or both of the test(s) be positive, a pre-defined testing algorithm will be followed to determine whether antibodies have been induced by the vaccine or whether the volunteer has become infected with HIV.<br>HIV testing at additional time points may be performed at the discretion of the volunteer and Investigator as medical or social circumstances arise.
**STATISTICAL CONSIDERATIONS:**	Collected data will be identified only by a study number. An interim analysis of grouped data will be carried out without unblinding the study to investigators or volunteers. At the end of the study, a full analysis will be prepared according to a pre-specified statistical analysis plan. Safety and tolerability will be addressed by examining the overall rates of adverse events and serious adverse events that might be associated with vaccination and the number of volunteers who experience these events. All clinical and routine laboratory data will be included in the safety analysis. Immunogenicity analyses will be performed in all volunteers who have received any vaccinations. Volunteers will be classified as responders or non-responders based on the results of the ELISPOT assay.

Table of Contents

ABBREVIATIONS 10

1.0. Sponsor Contact information 11

2.0 SIGNATURE PAGE 12

3.0 INTRODUCTION AND BACKGROUND 13

3.1 Viral Vector Vaccines 14
3.1.1 The Investigational Product MVA (ADMVA) vaccine 14
3.1.2 Pre-clinical studies with MVA vaccines 15
3.1.2.1 Pre-clinical studies with ADMVA 16
3.1.3 Human trials with MVA vaccines 17
3.1.4 Human trials with ADMVA 18
3.1.5 Human trials with other recombinant vaccinia and pox-virus based vaccines 18
3.1.6 Containment and transmission of MVA live vector vaccine 19

4.0 STUDY OBJECTIVES 19

4.1 Primary Objectives 19

4.2 Secondary Objectives 19

5.0 Study endpoints and study DESign 19

5.1 STUDY ENDPOINTS 19
5.1.1 Primary Endpoints 19
5.1.2 Secondary Endpoints 19

5.2 STUDY DESIGN 20
5.2.1 Duration of the Study 20
5.2.2 Study Population 20
5.2.3 Inclusion Criteria 21
5.2.4 Exclusion Criteria 21
5.2.5 Recruitment of Volunteers 23

6.0 STUDY VISITS 23

6.1 Screening Visit 23

6.2 Vaccination Visit Procedures 24

6.3 Post-Vaccination Follow-up Visits 25

6.4 Additional Follow-up Visits 25

UNCONTROLLED COPY

6.5 Unscheduled Visits 25

6.6 Final Visit/Early Termination Visit 26

7.0 Study PROCEDURES 26

7.1 Informed Consent 26

7.2 Medical History and Physical Examination 26

7.3 HIV Testing and Counseling 27

7.4 Pregnancy Avoidance Counseling 27

7.5 Blood Collection and Shipment 27

7.6 Reimbursement 27

7.7 Randomization and Blinding 27

7.8 Unblinding Procedure 28

8.0 INVESTIGATIONAL PRODUCT 28

8.1 Description 28

8.2 Shipment and Storage of Investigational Product 29

8.3 Dispensing and Handling of Investigational Product 29

8.4 Accountability and Disposal of Used and Unused Investigational Product 29

9.0 ASSESSMENTS 29

9.1 Safety Assessments 29
9.1.1 Local Reactogenicity Events 29
9.1.2 Systemic Reactogenicity Events 29
9.1.3 Other Adverse Events 30
9.1.4 Concomitant Medications: 30
9.1.5 Safety Laboratory Parameters 30
9.1.7 HIV Antibody Test 31

9.2 Immunogenicity Assessments 31
9.2.1 Antibody responses 31
9.2.2 Cellular Responses 31
9.2.3 PBMC, Serum and Plasma Storage 32

9.3 Other Assessments 32
9.3.1 CD4 and CD8 T cell count 32
9.3.2 HLA Typing 32
9.3.3 Anti-MVA Antibodies 32

10.0 ADVERSE EVENTS 32

UNCONTROLLED COPY

10.1. Definition 32

10.2. Assessment of Severity of Adverse Events 33

10.3 Serious Adverse Events 33

10.4 Relationship to Investigational Product 34

10.5 Reporting Serious Adverse Events 35

10.6 Clinical Management 36

10.7 Pregnancy 36

10.8 Evaluation for Intercurrent HIV Infection 36

11.0 Management of HIV issues during and following study 36

11.1 HIV Testing 36

11.2 Social Discrimination as a Result of an Antibody Response to Investigational Product 37

11.3 HIV Infection 37

11.3.1 Counseling 37

11.3.2 Referral for Support and/or Care 37

12.0 DISCONTINUATION OF VACCINATIONS AND/OR WITHDRAWal FROM STUDY 38

12.1 Discontinuation of Further Vaccinations 38

12.2. Follow up after discontinuation of further vaccinations 38

12.3 Withdrawal from the Study (Early Termination) 39

12.4 Follow-up after withdrawal from the study (Early Termination) 39

13.0 DATA HANDLING 39

13.1 Data Collection and Record Keeping at the Study Site 39

13.2 Data Collection and Transfer at the IAVI Core Laboratory 40

13.3 Data Entry at the Study Site 40

13.4 Data Handling and Analysis 40

14.0 STATISTICAL CONSIDERATIONS 40

14.1 Sample Size 40

14.2 Statistical Power and Analysis 41

15.0 QUALITY CONTROL AND QUALITY ASSURANCE 42

UNCONTROLLED COPY

16.0 DATA AND BIOLOGICAL MATERIAL 42

17.0 ADMINiSTRATIVE STRUCTURE 42

17.1 Trial Steering Committee (TSC) 42

17.2 Data and Safety Monitoring Board (DSMB) 43
17.2.1 Content of interim review 43
17.2.2 Indications for discontinuation of vaccinations in all volunteers 43

18.0 INDEMNITY 43

19.0 PUBLICATION 43

20.0 ETHICAL CONSIDERATIONS 43

REFERENCES: 45

APPENDIX A: SCHEDULE OF PROCEDURES 49

APPENDIX B: ADVERSE EVENT SEVERITY ASSESSMENT TABLE 50

APPENDIX C: SAMPLE INFORMED CONSENT FORM 57

UNCONTROLLED COPY

ABBREVIATIONS

Abbreviation	Term
AE	Adverse Event
AIDS	Acquired Immunodeficiency Syndrome
ALT	Alanine-Aminotransferase
AST	Aspartate-Aminotransferase
CFC	Cytokine Flow Cytometry
CMI	Cell Mediated Immunity
CTL	Cytotoxic T Lymphocyte
DCC	Data Coordinating Centre
DSMB	Data Safety Monitoring Board
DNA	Deoxyribonucleic Acid
ELISA	Enzyme Linked Immunosorbent Assay
GCP	Good Clinical Practice
HIV	Human Immunodeficiency Virus
HLA	Human Leukocyte Antigen
IAVI	International AIDS Vaccine Initiative
ICH	International Conference on Harmonization
MVA	Modified Vaccinia Ankara
PCR	Polymerase Chain Reaction
Pfu	Plaque Forming Units
PBMC	Peripheral Blood Mononuclear Cells
SAE	Serious Adverse Event
SIV	Simian Immunodeficiency Virus
SOM	Study Operations Manual
TSC	Trial Steering Committee

UNCONTROLLED COPY

1.0. SPONSOR CONTACT INFORMATION

Study Sponsor

International AIDS Vaccine Initiative (IAVI)
110 William Street, 27th Floor
New York, NY 10038-3901 USA

Medical Monitor

Soe Than, MD, PhD
Medical Project Director
International Aids Vaccine Initiative (IAVI)
110 William Street, 27th Floor
New York, NY 10038-3901 USA

Sponsor Medical Expert

Patricia Fast MD, PhD
Director, Medical Affairs
International AIDS Vaccine Initiative (IAVI)
110 William Street, 27th Floor
New York, NY 10038-3901, USA

IAVI Core Laboratory

Professor Frances Gotch
Department of Immunology,
Faculty of Medicine, Imperial College
Chelsea and Westminster Hospital
369 Fulham Road
London SW10 9NH
United Kingdom

UNCONTROLLED COPY

2.0 SIGNATURE PAGE

The signatures below constitute the approval of this protocol and the appendices and provide the necessary assurances that this study will be conducted in compliance with the protocol, GCP and the applicable regulatory requirement(s).

**Sponsor:**			
Signed:		Date:	
	Patricia Fast MD, PhD<br>Director, Medical Affairs, IAVI		
**Principal Investigator:**			
Signed:		Date:	
Name:			
Institute			

Instructions: The principal investigator at each study site will sign and date two copies of the protocol signature page indicating that he/she agrees to conduct the study in accordance with the protocol.

One copy of the original, signed protocol signature page will be returned to IAVI where it will be archived. The other copy of the original signed and dated protocol signature page must be filed in the investigator's site file.

UNCONTROLLED COPY

3.0 INTRODUCTION AND BACKGROUND

Despite two decades of effort against it, the global HIV-1 epidemic continues to plague humanity. In the face of such an unprecedented medical challenge, the scientific community has made important advances in the fields of virology, immunology and pharmacology. Nonetheless, it has proven extremely difficult both to contain the spread of infection around the world, and to prevent disease progression in most infected individuals. Since the beginning of the epidemic 65 million people have been infected. Globally over 42 million people are today living with HIV infection, with 5 million new infections acquired annually in 2002[1]. More than 25 million individuals have lost their lives to the disease since the beginning of the pandemic. Three million people died of AIDS in 2002. Over 95% of new HIV infections occur in developing countries, with the majority of infections found in Sub-Saharan Africa and South East Asia. Of the 5% who have access to antiretroviral medication, a significant subset will be intolerant of available drugs because of adverse effects, and another subset will harbor drug-resistant viral variants. Though public health outreach can help slow the rate of HIV-1 transmission in certain regions, it is clear that a protective vaccine would represent the most satisfying solution to the global problem.

One particular subtype of HIV-1 appears to have achieved phylogenetic dominance. Subtype C viruses now account for over 50% of new HIV-1 infections in the world. In particular, this clade has ravaged much of sub-Saharan Africa, and is now seen to encroach into Indochina [2-4]. From India and Myanmar, subtype C has gained a foothold in the People's Republic of China, presumably via transmission among intravenous drug-users (IVDUs) in the southwestern part of the country. Yunnan province is especially burdened, with nearly half of the HIV-1 cases in all of China. According to the Yunnan Bureau of Health, the prevalence of HIV-1 infection among IVDUs in the province was 29% in 2000, and is expected to reach 40.7% in 2005. Five counties in Yunnan (Wenshan, Honghe, Dehong, Lingchang and Dali) have the highest prevalence rates, estimated at between 50 and 75% (Figure 1). HIV-1 subtype C has also spread to neighboring provinces such as Sichuan and Guangxi, and is additionally responsible for much of the infection in distant Xinjiang.

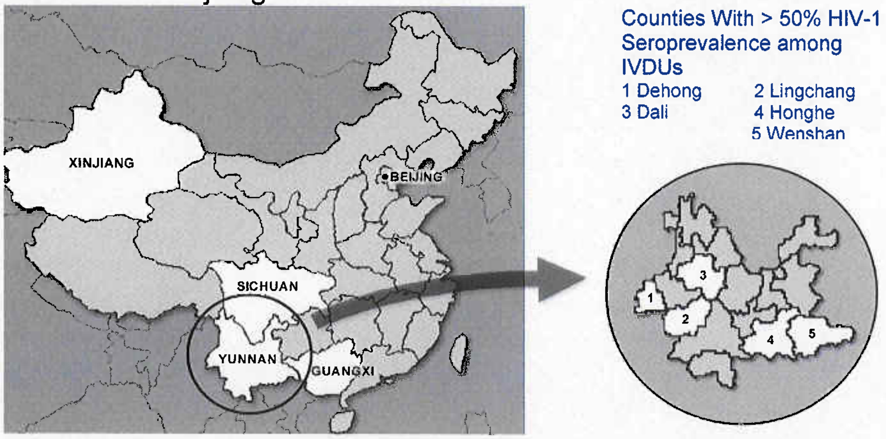

Figure 1: Epidemic regions in China

According to the Chinese Academy of Preventive Medicine, over 6 million people in the country are likely to be infected with HIV-1 by 2005. In recognition of and concern for the growing epidemic, Chinese public health authorities have established surveillance sites throughout the country, with a particular focus on Yunnan. By the end of 2000, there were a total of 43 such sites in the province, covering all 26 counties.

3.1 Viral Vector Vaccines

New viral vectors that may prove useful for HIV vaccines have been advanced in recent years. The best-studied vaccine vectors in humans are the poxviruses. Vaccinia virus engineered with HIV-1 genes has been shown to induce virus-specific cellular and humoral immune responses in immunized macaques, and the vaccine provided protection against simian immunodeficiency virus (SIV) infection, but only when immunization with such constructs was followed by boosting with recombinant proteins [5,6]. However, due to the potential for disseminated vaccinia infections in immunosuppressed individuals[7], there is a reluctance to use conventional vaccinia virus as a vector system in large clinical trials, where unsuspected HIV infection may occur. Particular attention has therefore focused on poxviruses with limited or no in vivo replicative capacity, such as canary pox, fowl pox and Modified Vaccinia Ankara (MVA). MVA is a highly attenuated strain of vaccinia virus that has undergone 570 passages in primary chicken embryo fibroblasts (CEF) and has genomic deletions that reduce its pathogenicity in mammalian cells[8]. MVA does not complete an entire replication cycle in human cells[8] but does initiate protein synthesis and thus elicits an immune response[9,10]. MVA was shown to be avirulent in immunosuppressed animals and, most importantly, to be safe with no reported adverse effects when used in 120,000 humans at the end of the smallpox eradication campaign[8,11-13].

A total of 540,824 military personnel were vaccinated with small pox (vaccinia) vaccine from December 2002 through December 2003. Myopericarditis developed in 67 of the vaccinees within two weeks following vaccination. Of these 67 vaccinees, 57% had ECG changes, however, nearly all patients had resolution of chest pain on follow-up. It has been concluded that myopericarditis should be considered in patients with chest pain within 30 days after small pox vaccination[14-15]. The experimental MVA vaccine in this study is related to the small pox vaccine and it is unknown if it could cause the same side effects as small pox vaccine. However, there have been no vaccine related cardiac symptoms reported among over 200 subjects who have been vaccinated with MVA-vectored HIV vaccine so far.

3.1.1 The Investigational Product MVA (ADMVA) vaccine

The multigenic recombinant ADMVA is designed as a homologous booster, corresponding to the plasmid DNA priming vaccine for HIV-1 subtype C. The HIV-1 structural genes (*env, gag, pol*) and regulatory genes (*nef, tat*) are encoded in the construct. The genes used in the vaccines were derived from a clade C strain, (Circulating Recombinant Form 007, or $HIV_{CHN.AD}$, which also contains segments of clade B) that is the dominant subtype in Yunnan

Province.

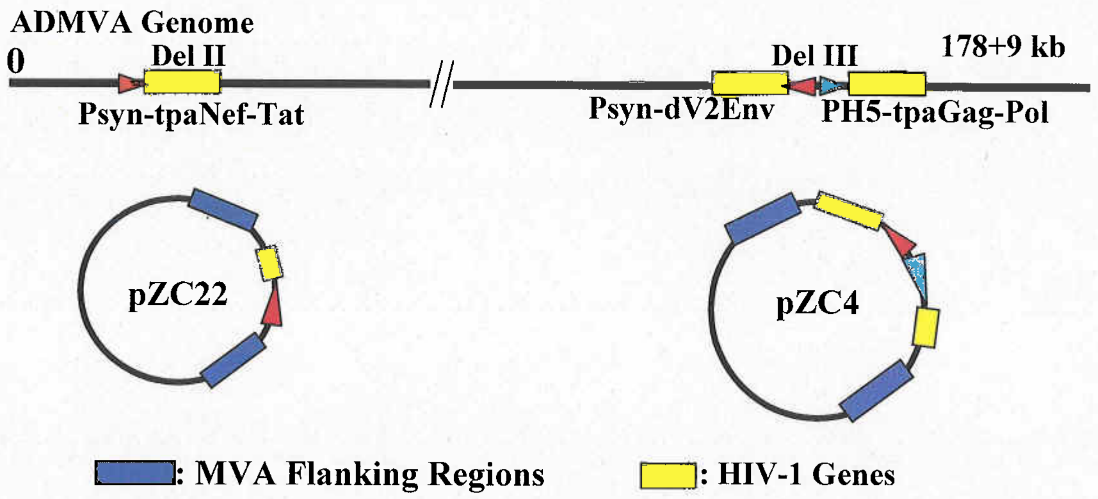

Figure 2. Genomic map of ADMVA. Engineering the multigenic recombinant with shuttle plasmids pZC22 and pZC4.

The five HIV-1 genes were originally characterized in the context of plasmid vectors for expressive potential, safety and immunogenicity. The multigenic recombinant, termed ADMVA was generated by homologous recombination between CEF cells infected with recombinant MVA clone ZC4PCRE11/12 (above) and the shuttle plasmid pZC22 that directed insertion of the tPA modified *nef-tat* fusion gene into MVA deletion II, more than 120kbp upstream of the del III region (Figure 2).

The Aaron Diamond AIDS Research Center (ADARC) laboratory used the parental strain at passage 585 to construct the subject multigenic recombinant MVA. The CEF used for propagation of the recombinant virus at ADARC are documented as being derived from SPAFAS eggs from certified flocks. The lots of FBS and trypsin used throughout the construction and characterization at ADARC are documented as derived from animal sources originating in BSE-free countries.

The first stage recombinant, expressing only DV2*env* and *gag-pol* genes, underwent 11 rounds of foci selection and purification prior to the final homologous recombination yielding the complete multigenic recombinant termed "ADMVA". ADMVA was further subjected to 9 rounds of foci selection and purification till clone AD27-5 was selected. Clone AD27-5 has undergone 5 additional passages in the random expansion of this seed stock. Vials from this frozen stock have been sent to the manufacturer of clinical batches for further passaging and establishment of a Master Seed Virus and Working Seed Virus before initiating production.

3.1.2 Pre-clinical studies with MVA vaccines

The immunogenicity of the MVA vaccine in animals is well documented. Recombinant MVA vector vaccines elicit SIV-specific CTL responses in rhesus monkeys[16,17] and mice[20], decrease plasma viraemia and increase survival[19,21] after challenge with pathogenic SIV. Several studies in macaques have demonstrated that, although CTL responses were detected following immunization with MVA-SIV recombinants, no animals were completely protected from infection upon challenge with pathogenic SIV. However, immunized animals had lower virus loads and

UNCONTROLLED COPY

prolonged survivals compared with control animals that received only non-recombinant MVA[17,19]. Finally, Ourmanov et al, have shown that the recombinant MVA-SIV also is a promising vector to prime an anamnestic neutralizing antibody response following the challenge with SIV[20].

The safety of MVA was studied in immune-suppressed macaques. Eight macaques were immunized with MVA by three different routes (intradermally, intramuscularly, intranasally) after immune-suppression by total body irradiation, anti-thymocyte globulin treatment or measles virus infection. No clinical, hematological or pathological abnormalities related to MVA inoculation were observed during a 13-day follow up period.

3.1.2.1 Pre-clinical studies with ADMVA

Preclinical immunogenicity studies demonstrated that homologous HIV-1 subtype C peptide-specific CTL responses were induced against all five HIV immunogens in BALB/c mice immunized with the multivalent recombinant ADMVA strain. Furthermore, ADMVA induced CMI responses regardless of the route of administration used in mice, and consistently strong responses were observed using the intramuscular route intended for clinical use. Despite strong MVA specific T-cell responses after a single immunization with ADMVA, mice demonstrated a boosted HIV-specific CMI response after a second immunization with ADMVA administered 3 weeks post priming response. The recombinant MVA vaccine elicited comparable CTL responses in two different strains of mice. Humoral immune responses were also observed when anti-gp120 and anti-gag antibody titers were measured. When the IgG subclasses of the resultant anti-gp120 antibody responses were compared, ADMVA elicited a balanced Th1 and Th2 response in BALB/c mice as shown by the comparable IgG1 and IgG2a *env*-specific antibody titers.

The systemic toxic potential of ADMVA HIV vaccine to Crl:CD-ITM(ICR)BR, mice was assessed by repeat intramuscular injections (total of 4 injections at Days 1, 22, 43 and 64) administered at three week intervals. Two groups of male and female mice received ADMVA at dosages of 10^6 or 10^7 pfu per mouse, and the third group (control) received the vehicle. Based upon the results of this study, repeated dosing with the ADMVA HIV Vaccine at 1 x10^6 and 1 x 10^7 pfu was well tolerated without evidence of systemic toxicity. Treatment related findings were limited to expected changes related to stimulation of the immune system by a vaccine e.g. transient inflammation/local reactivity at the injection site, slight increases in immunoglobulin and immune cells, and gross and microscopic changes in primary and secondary immune organs. Inflammation occurring at the most recent injection site allied to the prominent germinal centers/increased cellularity of the draining lymph nodes showed complete and rapid recovery. There were neither any treatment-related deaths, nor treatment-related effects noted on clinical signs, bodyweight, food intake or at the ophthalmic or blood chemistry examinations in this study. Higher white blood cell counts were observed mainly among treated male groups. Microscopically, an increase in the germinal center development/cellularity of the paracortex noted in the draining lumbar lymph nodes of mice sacrificed, both indicated at necropsy 72 h post final injection and absent at recovery. The macroscopic examination revealed slightly higher than control spleen weights, often associated with spleen enlargement, at the 10^7 pfu dose group with diminished incidence at the recovery necropsy. Enlargement of the lymph nodes draining the injection site was observed macroscopically among females treated at 10^7 pfu at 48-72 h post final injection.

Another GLP study was conducted to determine the potential toxicity and local tolerability of the ADMVA vaccine at a dose level of 2.5 x 10^8 pfu in 0.5 mL following repeated intramuscular administration (total of 3 injections at Days 1, 22 and 43) in rabbits. The dosage used in this

UNCONTROLLED COPY

study represents administration of one full human dose of the highest clinical dose level proposed for clinical use. The control group received sterile vehicle (Tris/NaCl buffer). Consistent with the safety assessments in CD-1 mice, no treatment related changes in mortality, clinical signs of toxicity, body weight changes, or overall food consumption were observed in rabbits treated with AMDVA vaccine during the in-life phase of study. There were no effects noted on coagulation or hematology parameters and no related ocular findings. Detailed observations of injection sites indicated no ADMVA-related effects upon the Draize scores for erythema and edema. Slight increases in serum globulin levels and inflammatory changes localized to injection sites were observed microscopically. The inflammatory changes had fully resolved at the Day 1 injection site by Day 46. These changes were attributed to the immunogenic effect of the vaccine. Evidence of specific anti-gp120 antibodies was observed in all animals within the ADMVA treatment group, as tested by indirect ELISA at laboratories of the Aaron Diamond AIDS Research Center, NY.

Further details regarding these studies can be found in the Investigator's Brochure.

3.1.3 Human trials with MVA vaccines

During the small pox eradication campaigns, over 120, 000 individuals were given the MVA without reports of safety problems[13,21,.22].

More recently, recombinant MVA constructs expressing tumor antigens (HPV and MUC1) have been administered in more than 50 cancer patients (for MVA-MUC1-IL2: breast, lung, prostate cancer patients at all disease stages; for MVA-HPV: cervical cancer patients at stages 1, 2, CIN3 and 4) in Europe and the United States[23]

The safety study of the recombinant MVA E2A was performed in 200 female patients in Mexico, having only papillomavirus infection but no lesion. The MVA E2 did not present any concerning pattern of adverse effects in these patients[24]

Several clinical studies have recently been reported using recombinant MVA vaccine expressing a synthetic malaria parasite gene, designated MVA ME-TRAP[25-27]. Volunteers in the UK and The Gambia have received this experimental vaccine, which has proved safe at a high dose of 15 x10^7 pfu, given as two intradermal injections, either alone or as a heterologous boost following DNA ME-TRAP priming[25]

A research program with the aim of developing recombinant vaccines for the prevention of HIV/AIDS, based on induction of a CTL response, has been conducted by the UK Medical Research Council at the Weatherall Institute for Molecular Medicine (WIMM), Oxford, UK. These vaccines are based on plasmid DNA and recombinant modified vaccinia virus Ankara (MVA) and are intended for use in heterologous DNA prime/MVA boost combination.

The first two HIV vaccines (DNA.HIVA and MVA.HIVA) developed by the WIMM encode a range of viral antigens contained within a synthetic polyepitope gene. The viral sequences contained in HIVA are derived from HIV-1 clade A, the predominant HIV clade in Kenya[27]. In a clinical development program sponsored by the International AIDS Vaccine Initiative (IAVI) the safety, effectiveness and suitability of these DNA/MVA vaccines are currently under evaluation at clinical sites in the Europe and Africa.

Results from one of those studies were reported recently[29]. In this study safety and immunogenicity of the vaccine was evaluated in healthy volunteers. Eighteen volunteers were treated with either 100ug or 500ug DNA.HIVA intramuscularly on days 0 and 21. Eight

UNCONTROLLED COPY

volunteers were intradermally vaccinated with $5x10^7$ pfu MVA.HIVA. Nine volunteers who were treated with either 100ug or 500ug DNA.HIVA received $5x10^7$ pfu MVA.HIVA 9-14 months later as prime boost regimen. Both DNA and the MVA vaccine alone and in a DNA prime-protein boost combination were safe and induced HIV specific responses (measured by IFN-γ ELISPOT) in 14 out of 18, seven out of eight and eight out of nine volunteers respectively. In MVA alone arm small reactions at the site of intradermal injection; signs included erythema, induration and very small (1-2mm) blister like lesions that never coalesced or broke which subsided in 14 days were reported. There were generally minimal systemic symptoms reported except one subject developed a febrile illness with vomiting. However, no similar reaction was observed in their subsequent studies in 100 volunteers (200 immunizations) with same MVA.HIVA[29].

In several trials where MVA.HIVA was administered intradermally at a dose of 5×10^7pfu either alone or as a heterologous boost following DNA.HIVA priming, the interim results showed MVA.HIVA to be generally safe and well-tolerated although erythema and induration often reached a degree classified as moderate or severe (1.5-3.0cm=moderate;>3.0cm=severe) when administered intradermally.

For further details refer to investigator brochure.

3.1.4 Human trials with ADMVA

ADMVA vaccine has not been tested in any clinical trials.

3.1.5 Human trials with other recombinant vaccinia and pox-virus based vaccines

Several different vaccinia vectors expressing different HIV genes have been tested in Phase 1 trials in humans. The safety and immunogenicity of TBC-3B HIV, a vaccinia based HIV vaccine, was shown in clinical trials in vaccinia immune and vaccinia naïve individuals. A total of 36 volunteers received TBC-3B, either by scarification, intradermal or subcutaneous administration. TBC-3B was well tolerated and no Serious Adverse Events were reported in trial [30].

The safety and immunogenicity of recombinant vaccinia virus (HIVAC-1e) vaccine was evaluated in vaccinia naïve healthy adults. Two doses of either 10^6 or 10^7 pfu/mL were given by bifurcated needle puncture. Vaccination with HIVAC-1e was safe and induced both humoral and cell-mediated immunity [31,32].

Recombinant vaccinia virus expressing a transmembrane glycoprotein and cytokines has been used in therapeutic vaccine trials in patients with breast, lung and prostate cancer. The vaccines were well tolerated, with no Grade 3 or 4 toxicities seen. Common Grade 1 and 2 toxicities include: fever, fatigue and erythema at vaccine site, light-headedness, and myalgia. No vaccine virus shedding was detected during the initial clinical trial (unpublished data from Transgene Inc., Strasbourg, France).

Several recombinant canary pox vaccines bearing gag, protease and/or env genes (ALVAC) have been tested in over 2000 volunteers. Canary pox, like MVA, is unable to replicate in human cells [34-40]. The HIVNET 007 trial in Uganda enrolled 40 volunteers, 20 of whom received a canary pox vaccine encoding env, gag and protease genes [40]. The recombinant canary pox vaccines have proved safe and have induced T-cell responses but little antibody [33-39].

UNCONTROLLED COPY

3.1.6 Containment and transmission of MVA live vector vaccine

The MVA live vector does not replicate in human cells and therefore is not transmissible between individuals.

4.0 STUDY OBJECTIVES

4.1 Primary Objective

- To evaluate the safety of ADMVA administered three times, intramuscularly at 3 dosage levels.

4.2 Secondary Objective

- To evaluate the immunogenicity of ADMVA administered three times, intramuscularly at 3 dosage levels.

5.0 STUDY ENDPOINTS AND STUDY DESIGN

5.1 STUDY ENDPOINTS

5.1.1 Primary Endpoints

Safety and Tolerability:

- The proportion of volunteers with severe local reactogenicity events (e.g. pain, tenderness, erythema/skin discoloration, skin damage [vesiculation/ulceration], induration, edema, formation of crust or scab)
- The proportion of volunteers with severe systemic reactogenicity events (e.g. fever, chills, headache, nausea, vomiting, malaise, myalgia, rash, arthralgia)
- The proportion of volunteers with severe other adverse events (including laboratory abnormalities)
- The proportion of volunteers with serious adverse events
- Proportion of volunteers with mild and moderate local and systemic reactogenicity events and adverse events

5.1.2 Secondary Endpoints

Immunogenicity:

- The proportion of volunteers with HIV-1 specific T- cell responses quantified by ELISPOT.
- The proportion of volunteers with HIV-1 specific T-cell responses quantified by cytokine flow cytometry (CFC).

- All immune responses will be evaluated for proportion of responders and the mean responses will be compared.

5.2 STUDY DESIGN

The study is a randomized, dose-escalating, double blind (with respect to active vaccine or placebo), placebo-controlled study.

Safety and tolerability of the ADMVA vaccine/placebo will be evaluated at least 14 days after the 12th volunteer in the lower dosage group receives the second injection before proceeding to the middle dosage group. Likewise, there will be an evaluation at least 14 days after the 12th volunteer in the middle dosage group receives the second injection before proceeding to the higher dosage group.

Table 5.2
Study Design

Group	Vaccine/ Placebo	Month 0*	Month 1*	Month 6*
Low	12/4	1X10^7pfu<br>ADMVA or<br>Placebo<br>IM	1X10^7pfu<br>ADMVA or<br>Placebo<br>IM	1X10^7pfu<br>ADMVA or<br>Placebo<br>IM
Middle	12/4	5X10^7pfu<br>ADMVA or<br>Placebo<br>IM	5X10^7pfu<br>ADMVA or<br>Placebo<br>IM	5X10^7pfu<br>ADMVA or<br>Placebo<br>IM
High	12/4	2.5X10^8pfu<br>ADMVA or<br>Placebo<br>IM	2.5X10^8pfu<br>ADMVA or<br>Placebo<br>IM	2.5X10^8pfu<br>ADMVA or<br>Placebo<br>IM
Total Number	36/12			

*ADMVA or Placebo given IM at the dose indicated

5.2.1 Duration of the Study

Volunteers will be screened up to 42 days before vaccination and will be followed for 18 months after the first vaccination.

5.2.2 Study Population

Approximately 48 healthy male or female adults 18-40 years of age (36 vaccine recipients, 12 placebo recipients) who meet all the eligibility criteria will be included in the study. An over-enrolment of up to 10% (approximately 5 additional volunteers) will be accepted in the study.

UNCONTROLLED COPY

5.2.3 Inclusion Criteria

1. Healthy adult males and females, as assessed by a medical history, physical exam, and laboratory tests;
2. Age of at least 18 years of age on the day of screening and no greater than 40 years on the day of first vaccination;
3. Willing to comply with the requirements of the protocol and available for follow-up for the planned duration of the study (screening plus 18 months);
4. In the opinion of the principal investigator or designee has understood the information provided. Written informed consent needs to be given before any study-related procedures are performed;
5. Willing to undergo HIV Testing and counseling, and receive HIV test results;
6. If sexually active female, using an effective method of contraception (combined oral contraceptive pill; injectable contraceptive; diaphragm; Intra Uterine Device (IUD); condoms; anatomical sterility in self or partner) from screening until at least 4 months after last vaccination. All female volunteers must be willing to undergo urine pregnancy tests at time points as indicated in the schedule of procedures (Appendix A);
7. If sexually active male, willing to use an effective method of contraception (such as condoms, anatomical sterility) from screening until 4 months after the last vaccination and will be advised not to get his partner pregnant.

5.2.4 Exclusion Criteria

1. Confirmed HIV-1 or HIV-2 infection;
2. Reported high-risk behavior for HIV infection defined as:

   Within 6 months before vaccination, the volunteer has:
   - Had unprotected vaginal or anal sex with a known HIV infected person or with a casual partner (i.e., no continuing established relationship).
   - Engaged in sex work for money or drugs
   - Used injection drugs (illicit), or
   - Acquired an STD;
3. Any clinically significant abnormality on history or examination including history of immunodeficiency or autoimmune disease; use of systemic corticosteroids, immunosuppressive, antiviral, anticancer, or other medications considered significant by the trial physician within the last 6 months;
4. Any clinically significant acute or chronic medical condition requiring care of a physician (e.g., diabetes, coronary artery disease, rheumatologic illness, malignancy, substance abuse) that in the opinion of the investigator would preclude participation;
5. Any of the following abnormal laboratory parameters listed below:
   - Hemoglobin: <9.0 g/dL
   - Absolute Neutrophil Count (ANL): ≤ 999/mm^3

UNCONTROLLED COPY

- Absolute Lymphocyte Count (ALC): ≤ 500/mm^3
- Platelets: ≤ 90,000 ≥ 550,000/mm^3
- Creatinine: > 1.4 x ULN
- AST: >3.0 x ULN
- ALT: >3.0 x ULN
- Urine dipstick: blood = 2+ or more (except in menstruating females); protein = 2+or more
- Cardiac troponin I: > ULN;

6. Confirmed diagnosis of hepatitis B (surface antigen, HbsAg); hepatitis C (HCV antibodies) or active syphilis;

7. If female, pregnant or planning a pregnancy within 4 months after last vaccination or lactating;

8. Receipt of a live attenuated vaccine (other than influenza) within 30 days or other vaccine within 14 days of vaccination;

9. Receipt of blood transfusion or blood products 6 months prior to vaccination;

10. Participation in another clinical study of an investigational product currently or within past 12 weeks or expected participation during this study;

11. Receipt of another experimental HIV vaccine at any time;

12. History of severe local or systemic reactogenicity to vaccination or history of severe allergic reactions;

13. Major psychiatric illness including any history of schizophrenia or severe psychosis, bipolar disorder requiring therapy, suicidal attempt or ideation in the previous 3 years;

14. In the opinion of the investigator, unlikely to comply with protocol;

15. ECG with clinically significant findings or features that would interfere with the assessment of myo/pericarditis including:
    - conduction disturbance (atrioventricular or intraventricular condition, left or right bundle branch block, AV block of any degree, or QTc prolongation)
    - repolarization (ST segment or T wave) abnormality
    - significant atrial or ventricular arrhythmia
    - frequent atrial or ventricular ectopy (e.g. frequent premature atrial contractions, 2 premature ventricular contractions in a row)
    - ST elevation consistent with ischemia
    - evidence of past or evolving myocardial infarction;

16. History of, or known active cardiac disease including:
    - previous myocardial infarction (heart attack)
    - angina pectoris
    - congestive heart failure
    - valvular heart disease including mitral valve prolapse
    - cardiomyopathy
    - pericarditis
    - stroke or transient ischemic attack

UNCONTROLLED COPY

- chest pain or shortness of breath with activity (such as walking up stairs)
- other heart conditions under the care of a doctor;

17. Have 3 or more of the following risk factors:
    - high blood pressure diagnosed by a doctor
    - high blood cholesterol diagnosed by a doctor
    - diabetes or high blood sugar diagnosed by a doctor
    - a first degree relative (for example, mother, father, brother, sister) who had a heart condition before the age of 50
    - smoke cigarettes now.

18. History of allergy to aminoglycosides (e.g. gentamycin)
19. History of severe allergy to eggs or egg products e.g., "rash or breathing difficulties"

5.2.5 Recruitment of Volunteers

Healthy adult male and female volunteers will be recruited through various means including, but not limited to, the following: information presented in community organizations, hospitals, colleges, and other institutions, and/or advertisements to the general public. Interested parties may be given telephone contact information and may be invited to attend a seminar where basic information about the study is provided. Individuals who are interested in participating may have a telephone interview, which would let them know if they might qualify for study participation. They may be invited to attend additional informational seminars, at which detailed information about the study and the requirements for participation would be provided. Potential volunteers will be given a site-specific consent information sheet (sample Informed Consent document in Appendix C). They will have the opportunity to ask any questions they might have and to talk to a research team member. If they are still interested and willing to participate, they will be invited for a screening visit. Other processes, such as one-on-one sessions with study team members, may occur during which counseling will be provided and full informed consent will be obtained.

6.0 STUDY VISITS

6.1 Screening Visit

During the Screening Visit, site personnel will answer any questions about the study. Written site-specific informed consent will be obtained prior to conducting any study procedures. To ensure informed consent, the principal investigator or designee will discuss the following processes and explanations individually with each volunteer:

1. Pre HIV-test counseling
2. Risk-reduction counseling including safe-sex counseling
3. That it is unknown whether or not the study vaccines will protect against HIV infection or disease and, if so, the extent of that protection
4. That, following vaccination, it may be possible that the volunteer will develop antibodies against HIV, which may produce a positive reaction in a routine HIV test, and that provisions have been made to distinguish between response to vaccine and HIV infection during and after the trial.

UNCONTROLLED COPY

5. That a sexually active volunteer should use a reliable form of contraception from screening, during the vaccination period until 4 months after the last vaccination.

If the volunteer consents to participate, site personnel will:

- Provide screening questionnaire to volunteer
- Perform HIV risk assessment
- Perform complete medical history (including concomitant medication)
- Perform a general physical examination including height, weight, vital signs (pulse, respiratory rate, blood pressure and temperature), examination of skin, respiratory, cardiovascular and abdominal systems, and an assessment of cervical and axillary lymph nodes.
- Perform ECG
- Collect blood and urine specimens for all tests as indicated in the Schedule of Procedures (Appendix A).
- Perform a pregnancy test for all female volunteers
- If volunteer has a history of small pox vaccination, this should be documented in clinic notes.

Screening laboratory test(s) may be repeated once at the discretion of the principal investigator or designee to investigate any isolated abnormalities.

If the screening visit occurs more than 42 days prior to date of vaccination, then study procedures for the screening visit must be repeated. However, the complete medical history may be replaced by an interim medical history and the Informed Consent form may be reviewed without signing again.

6.2 Vaccination Visit Procedures

Prior to the first vaccination, site personnel will:

- Answer any questions about the study;
- Review interim medical history (including concomitant medications);
- Review screening safety laboratory data;
- Review the Informed consent administered at screening visit with volunteer;
- Perform a directed physical examination including vital signs (pulse, respiratory rate, blood pressure and temperature), as well as an assessment of axillary lymph nodes and any further examination indicated by history or observation);
- Conduct pre HIV-test counseling;
- Collect blood and urine specimens for all tests as indicated in the Schedule of Procedures (Appendix A);
- Perform a pregnancy test for all female volunteers and obtain results prior to vaccination;
- Perform baseline assessment of the site of injection and evaluate and record any systemic symptoms;
- Administer vaccine (the preferred site of first administration is the deltoid muscle of the upper arm. It is suggested that alternating arms be used for each vaccination). A 1 inch 23 gauge needle and a 3.0 ml syringe will be used.

Complete instructions for the handling and administration of the investigational product are supplied in the Study Operations Manual (SOM).

UNCONTROLLED COPY

Volunteers will be closely observed for at least 30 – 45 minutes after each vaccination. Vital signs (pulse, respiratory rate, blood pressure and temperature) will be monitored at 30 – 45 minutes after each vaccination and recorded. Any local and systemic reactogenicity events, as well as any other event that occurs, will be recorded at 30 – 45 minutes. Volunteers will be asked to record any reactogenicity events that occur between Day 1 and Day 3. In the case where the events within 3 days are moderate or severe, the volunteer may be requested to visit the site for appropriate assessment and treatment (if required). On that visit the volunteer will be asked to record if events continue. Site staff will explain to the volunteer how to record reactogenicity events.

6.3 Post-Vaccination Follow-up Visits

Volunteers will receive a phone call from the study staff between the 2nd and the 4th day after each vaccination and volunteers will be asked to return to the clinic on Day 7 and Day 14 after the first vaccination and at Day 14 after the second and third vaccination visits. At the Day 7 and Day 14 visits the volunteers will be asked about any local and systemic reactogenicity or other adverse events.

The following will be conducted at this visit:

- Review of interim medical history and use of concomitant medications;
- If symptoms are present, perform a symptom-directed physical examination;
- Assess local and systemic reactogenicity as well as any other adverse events;
- Collect blood and urine specimens for all tests as indicated in the Schedule of Procedures (Appendix A);
- Perform ECG as indicated in the Schedule of Procedures (Appendix A).

6.4 Additional Follow-up Visits

Assessments and procedures will be performed according to the Schedule of Procedures (Appendix A). Post HIV-test counseling will be performed as the HIV-test results become available.

In case of adverse event(s), the volunteer will be assessed and followed up by the clinical team. Supplemental visit(s) for further investigation can be planned at the discretion of the principal investigator or designee. Supplemental visit(s) may be recommended if clinically indicated or to clarify observations.

6.5 Unscheduled Visits

Unscheduled visits may be performed at any time during the study for various reasons such as:

- For administrative reasons, e.g., the volunteer may have questions regarding study or may need to re-schedule a follow-up visit.
- To obtain laboratory test results from a previous visit.
- In the event that a volunteer having missed a scheduled visit presents to the study site
- For other reasons as requested by the volunteer or site investigator.

All unscheduled visits and all procedures performed at those visits must be documented in clinic notes.

UNCONTROLLED COPY

6.6 Final Visit/Early Termination Visit

Assessments and procedures will be performed according to the Schedule of Procedures (Appendix A).

7.0 STUDY PROCEDURES

7.1 Informed Consent

All volunteers will give their written consent to participate in the trial on the basis of appropriate information and with adequate time to consider this information and ask questions.

A volunteer's consent to participate must be obtained by having him/her sign, date and give time of signing an IRB-approved, site-specific informed consent form that is also signed and dated by a member of the trial team prior to initiation of study procedures. The signed and dated informed consent form will remain at the study site for verification. A copy of the signed and dated informed consent form will be offered to the volunteer.

Potential trial volunteers will be informed about the possible benefits and risks of the vaccine and that there may be unknown risks. The volunteer will have access to information about the procedures for obtaining medical treatment and/or compensation in the event of trial-related injuries resulting from their participation in this study. The volunteers will be made aware before consenting to participate that they are free to withdraw without obligation at any time and that such an action will not adversely affect any aspect of their social or legal benefits and they will not be denied access to available medical care for which they might otherwise be eligible. If any changes to the protocol are made that may affect the volunteer's decision to continue in the study, an amended informed consent will be provided to the volunteers. Volunteers will be notified if new safety information becomes available that may affect their willingness to continue in the study.

7.2 Medical History and Physical Examination

At the time of screening, a past medical history will be collected that will include details of any previous reactions to vaccination, history of epilepsy, and contraceptive practices. An interim medical history will be collected at time-points according to Schedule of Procedures (Appendix A).

A general physical examination will be conducted including weight, height, vital signs, and examination of skin, respiratory, cardiovascular, central nervous and abdominal systems, as well as an assessment of cervical and axillary lymph nodes. At the time of vaccination and at select time-points thereafter, general and/or directed physical examinations will be performed according to Schedule of Procedures (Appendix A). A directed physical examination will include weight, vital signs, examination of injection site, as well as an assessment of axillary lymph nodes and any further examination indicated by history or observation.

UNCONTROLLED COPY

7.3 HIV Testing and Counseling

Study personnel will assess volunteers for past and current risk of HIV infection and counsel them prior to collecting blood for an HIV test. Study personnel will perform post HIV-test counseling as indicated in the Schedule of Procedures (Appendix A). The counseling process will include information on HIV, safe sex practices and risk reduction. The objective of counseling is to ensure that volunteers have sufficient knowledge about HIV infection to understand for the purpose of the test, the implications of a positive or negative result and the standard of care available locally for HIV infection. Volunteers will be told that it is possible to have a false positive HIV test result due to a response to the vaccine. Additionally, risk reduction counseling, including safe-sex counseling, will be provided during the study to reinforce low-risk behavior.

7.4 Pregnancy Avoidance Counseling

Study personnel will counsel volunteers at screening and on the day of each vaccination about the importance of prevention of pregnancies and the use of condoms, as well as other effective family planning methods.

7.5 Blood Collection and Shipment

Venous blood will be collected at visits, usually from the antecubital fossa, according to the Schedule of Procedures (Appendix A). At no time will the total volume of blood collected exceed 550ml over an 8 week period. All specimens will be handled according to site-specific SOPs and processed according to the IAVI Core Laboratory SOPs.

Frozen peripheral blood mononuclear cells (PBMC) will be shipped to the IAVI Core Laboratory, London, where ELISPOT and CFC assays will be performed. PBMC, plasma and serum samples will also be shipped to the Core Laboratory for repeat testing and quality control and for future assays related to HIV vaccine research and development (this is subject to approval by the Trial Steering Committee and IRB/IEC). Only a code number will identify the samples. A portion of the blood collected at base line and the blood collected for immunogenicity assays at month 9 will be processed at ADARC for research purpose.

7.6 Reimbursement

Volunteers will be reimbursed to cover their travel expenses, as well as child care and time lost from gainful employment. Reimbursement will be outlined in the site-specific informed consent forms.

7.7 Randomization and Blinding

The randomization schedule will be prepared by the statisticians at the Data Coordinating Center (the EMMES Corporation). The randomization list will be sent to a packaging contractor for labeling and packaging of study vaccine and placebo in a double blind fashion.

Study site staff and volunteers will be blinded only with respect to the allocation of placebo or vaccine to volunteers. The procedure for maintaining the blind is described in the Study Operations Manual. Blinding will not apply to the assignment of dosage levels (low, middle or high dose). Volunteers will be informed about their group assignment once the data analysis is completed.

UNCONTROLLED COPY

7.8 Unblinding Procedure

Unblinding of an individual volunteer is indicated only in the event of a medical emergency where the clinical management/medical treatment of the volunteer would be altered by knowledge of the group assignment.

A two-layered tear off sheet will be kept in a sealed envelope in a secure location at the site. In the unlikely circumstance that a volunteer's group assignment needs to be revealed (i.e., unblinded) to assist in the clinical management of a Serious Adverse Event, the principal investigator or designee (after consultation with the IAVI Director of Medical Affairs) will tear off the slip from this sheet to reveal the code for that one individual. The reasons for unblinding should be documented and the EMMES Corporation should be notified. The tear off sheet must be returned to the EMMES Corporation at the end of the trial. Procedures and contact numbers for unblinding are outlined in the Study Operations Manual.

8.0 INVESTIGATIONAL PRODUCT

8.1 Description

The ADMVA vaccine and placebo are manufactured under cGMP conditions by Impfstoffwerk Dessau-Tornau GmbH (IDT) in Rosslau, Germany.

ADMVA contains Clade C HIV env/gag-pol and nef/tat fusion genes encoded in a single recombinant MVA. ADMVA is formulated in a sterile buffered solution containing 10mM Tris (hydroxymethyl)-amino methane, 140mM sodium chloride at pH 7.7 ±-0.5. ADMVA is supplied as a frozen suspension that is opaque, whitish in appearance when thawed at room temperature.

The Placebo is 10mM Tris (hydroxymethyl)-amino methane, 140mM sodium chloride at pH 7.7 ±-0.5. Both the investigational vaccine and the placebo are supplied as a single dose vial presented in a 2 ml glass vial with rubber injection stopper and an aluminum flip-top flange.

The description of the investigational products and placebo are listed - below in Table 2.

Table 8.1: Investigational Product

Vaccine/ Placebo	Dosage Level / 0.5 ml	Total Volume in Vial (ml)	Total Injected Volume (ml)	Route of Administration
ADMVA	$1x10^7$ pfu	0.7ml	0.5ml	IM
	$5x10^7$ pfu	0.7 ml	0.5ml	IM
	$2.5x10^8$ pfu	0.7 ml	0.5ml	IM
Placebo	NA	0.7 ml	0.5ml	IM

IM=Intramuscular

UNCONTROLLED COPY

8.2 Shipment and Storage of Investigational Product

Authorization to ship the investigational product to the site will be provided in writing by IAVI, upon confirmation that all required critical documents for shipment authorization are collected. The vaccines will be shipped to the respective sites on dry ice. The ADMVA vaccine and placebo are stored at -80°±10°C.

8.3 Dispensing and Handling of Investigational Product

The Investigational Product will be dispensed as specified in the Study Operations Manual. Designated site personnel will ensure that the allocation number on the vial matches the allocation number assigned to the volunteer.

The Investigational Product will be used as supplied by the manufacturers with no further preparation. Holding the vials in the hand thaws the Investigational Product. The vials must not be shaken. The required volume will be drawn into the appropriate syringe.

8.4 Accountability and Disposal of Used and Unused Investigational Product

All used vials will be returned to the vaccine dispenser or pharmacy according to site-specific SOPs at the end of each vaccination visit. The date, allocation number and location of storage of the returned vials will be recorded on a log. During the trial, the investigational product accountability form, the dispensing log and the log of returned vials will be monitored. At the end of the trial, the monitor will check all used and unused vials against the inventory before the vials are returned to the sponsor or destroyed on site upon written instructions from the sponsor

9.0 ASSESSMENTS

9.1 Safety Assessments

Data on local and systemic reactogenicity will be collected by structured interview, using specific questions. Data on other adverse events will be collected with an open-ended question. All data will be recorded on the appropriate source documents.

9.1.1 Local Reactogenicity Events

The presence of local reactogenicity will be assessed at the time points specified in the Schedule of Procedures (Appendix A).

Local reactogenicity events (pain/tenderness, erythema, edema, skin damage [vesiculation/ulceration], induration, formation of crust scab or scar) will be assessed according to Appendix B, Adverse Event Assessment Severity Table.

9.1.2 Systemic Reactogenicity Events

The presence of systemic reactogenicity will be assessed at the time points specified in the Schedule of Procedures (Appendix A).

Vital signs (pulse, respiratory rate, blood pressure and temperature) will be measured prior to vaccination and 30-45 minutes post-vaccination by study staff and graded according to Appendix B, Adverse Event Grading Toxicity Table, and recorded. All medication required for treatment will be recorded.

UNCONTROLLED COPY

Fever, chills, headache, nausea, vomiting, malaise, fatigue, myalgia, arthralgia, shortness of breath, chest pain, palpitation, reduction in exercise tolerance, rash and allergic reaction will be recorded and assessed according to Appendix B, Adverse Event Assessment Severity Table.

9.1.3 Other Adverse Events

Other adverse events will be recorded following an open question to volunteers, with the dates of commencement and resolution and any medication required. All adverse events will be followed to resolution. Serious Adverse Events will be collected during the entire study period. They will be graded as indicated in Appendix B, Adverse Event Assessment Severity Table. For more information regarding adverse events refer to Section 10, Adverse Events.

9.1.4 Concomitant Medications:

During the study, information regarding concomitant medications and reasons for their use will be solicited from the study volunteers at each visit and recorded. Concomitant receipt of investigational products, including other HIV vaccines is prohibited during the study.

If clinically indicated, non-live vaccines (non-HIV) and immunoglobulins may be given up to 2 weeks before study vaccination(s) or after post-vaccination blood draw (i.e. 2 weeks after study vaccinations).

Live-attenuated vaccines (non-HIV) may be given 30 days before study vaccination(s) or after the post-vaccination blood draw. However, the study vaccination(s) should not be given if there are any continuing symptoms from recently administered non-HIV vaccines. In this situation, the site investigator should consult with IAVI Medical Monitor before administering the next study vaccination.

If the use of a short tapering (<2 weeks) of systemic corticosteroids is required, the study vaccinations may be continued after 4 weeks washout period provided that the medical condition requiring this therapy has completely resolved and in the opinion of both, the site investigator and IAVI Medical Monitor, the continuation of the study vaccinations will not jeopardize the safety of the volunteer. Volunteers requiring chronic (> 2 weeks) or long term systemic corticosteroids therapy will be considered case by case.

9.1.5 Safety Laboratory Parameters

Safety laboratory parameters will include:

- Hematology: complete blood count, differential and platelet count
- Clinical chemistry: liver function tests: aspartate transferase (AST), alanine transferase (ALT), total and direct bilirubin.
- Kidney function test: creatinine
- Dipstick test for protein, blood, glucose, ketones, esterase (leukocytes), and nitrite. If abnormalities are found on dipstick analysis for protein, blood, or leucocytes, urine microscopy will be performed.

The samples for these tests will be collected at the time points indicated in the Schedule of Procedures (Appendix A). In the event of an abnormal laboratory value, volunteers may be asked to have an additional sample collected at the discretion of the principal investigator or designee. Volunteers will be screened for syphilis, viral hepatitis (HBsAG, Anti-HCV), at the screening visit only.

UNCONTROLLED COPY

A urine pregnancy test for all female volunteers will be performed by measurement of Human Chorionic Gonadotrophin (β-HCG) at the time points indicated in the Schedule of Procedures (Appendix A). The results of the pregnancy test must be obtained prior to each study vaccination.

9.1.6 Cardiac Assessments

Testing for cardiac troponin I will be performed as specified in the Schedule of Procedures (Appendix A). Once a participant has been enrolled, any ECG suggestive of pericarditis or myocarditis or cardiac troponin result above the institutional upper limit of normal should be reported to the sponsor once the results are available, and repeated as soon as possible along with a CK-MB (creatine kinase-MB isoenzyme), an erythrocyte sedimentation rate (ESR), an additional ECG, referral to a cardiologist, and an Echocardiogram.

A 12-lead ECG will be performed as specified in the Schedule of Procedures (Appendix A).

9.1.7 HIV Antibody Test

Only volunteers who are not infected with either HIV-1 or HIV-2 at screening will be enrolled into the study.

HIV antibody tests will be performed at the time points indicated in the Schedule of Procedures (Appendix A).

9.2 Immunogenicity Assessments

9.2.1 Antibody responses

Binding and neutralizing assays will be performed as specified in the Schedule of Procedures (Appendix A).

9.2.2 Cellular Responses

Immunogenicity assays, including ELISPOT and cytokine flow cytometry (CFC), will be used to measure *in-vitro* T cell responses following stimulation by HIV-specific antigenic peptides. This will be conducted at various time points as indicated in the Schedule of Procedures (Appendix A). Selected T cell responses may be further characterized for HLA restriction and epitope specificity.

Immunological testing for responses to vaccine encoded HIV antigens will be performed at the IAVI Core Laboratory in accordance with IAVI standard operating procedures and standard reagents.

Serum antibodies and T-cell responses against MVA may be examined using ELISA, viral neutralization assays, ELISPOT assays and CFC at selected time points.

9.2.3 PBMC, Serum and Plasma Storage

Cryopreserved PBMC, plasma and serum taken from time points as indicated in the Schedule of Procedures (Appendix A) will be used for purposes of immunogenicity assays, quality control and for future assays related to HIV vaccine research and development. These samples will be archived and only a code will identify the samples. At each time point indicated in the Schedule of Procedures (Appendix A), using the procedure provided by the IAVI Core Laboratory, at least three and generally more than five vials of frozen peripheral blood mononuclear cells (PBMC), each containing approximately 1.5×10^7 PBMC, will be taken for primary immunogenicity analysis (ELISPOT, CFC) and/or quality control assays at the IAVI Core Laboratory. These samples will be shipped to IAVI core lab according to an agreed schedule. Laboratory personnel will be trained as necessary by the sponsor and provided with a written procedural manual pertaining to PBMC isolation, counting, freezing and shipping. The sponsor will also provide specific instructions on reagents.

The samples described in 9.2.2 and 9.2.3 will be shipped routinely from each site to the IAVI Core Laboratory. The immunological testing will be performed at the IAVI Core Laboratory in accordance with IAVI standard operating procedures and standard reagents.

9.3 Other Assessments

9.3.1 CD4 and CD8 T cell count

CD4 and CD8 T cell count (absolute and percentage) will be measured using blood sample collected according to schedule of procedures (Appendix A).

9.3.2 HLA Typing

HLA samples will be collected according to schedule of procedures (Appendix A). HLA typing will be performed on samples for all participants vaccinated at a dosage level, provided T-cell responses are detected at that dosage level. The results of tissue typing will be kept confidential and is only for the purpose of characterizing the T-cell immune response. Volunteers will not receive results of the HLA-typing.

9.3.3 Anti-MVA Antibodies

Serum antibodies against MVA may be examined at selected time points according to schedule of procedures (Appendix A).

10.0 ADVERSE EVENTS

10.1. Definition

An adverse event (AE) is any untoward medical occurrence during the course of the study in a volunteer who received investigational product and that does not necessarily have a causal relationship with the investigational product. An AE can therefore be any unfavorable or

UNCONTROLLED COPY

unintended sign (including an abnormal laboratory finding), symptom, or disease, temporally associated with the use of the study product whether or not related to the study products.

Whenever possible, the laboratory abnormalities should be considered in the context of the primary clinical diagnosis and reported as such (e.g. acute hepatitis with increased bilirubin, ALT, AST and GGT).

Any adverse event prior to the first injection will be reported as a pre-existing condition. All adverse events identified during the trial will be followed up until resolution or until the adverse event is judged by the principal investigator or designee to have stabilized. All vaccine related clinical events will be collected as part of the adverse event reporting to assess safety in the vaccinated recipients as compared to placebo recipients.

10.2. Assessment of Severity of Adverse Events

Assessment of severity of all AEs is ultimately the responsibility of the principal investigator. The following general criteria should be used in assessing the severity of adverse events as mild, moderate or severe:

- **Mild:** Mild discomfort; Minimal or no limitation of daily activities; Medical intervention not required;
- **Moderate:** Moderate discomfort; Some limitation of daily activities but able to work part-time or full-time with some assistance; may require minimal or no medical intervention;
- **Severe:** Severe discomfort; marked limitation of daily activities, unable to work; Requires medical intervention;

Criteria for grading the severity of specific adverse events and laboratory abnormalities are listed in Appendix B, Adverse Event Severity Table.

10.3 Serious Adverse Events

An adverse event is reported as a "Serious Adverse Event" by ICH Good Clinical Practice (ICH GCP) criteria if it:

- **Results in death,**
- **Is life-threatening,** Note: The term "life-threatening" in the definition of "Serious" refers to an event in which the participant was at immediate risk of death. It does not include a reaction that, had it occurred in a more severe form, might have caused death.
- **Results in persistent or significant disability/incapacity,** Note: the term "persistent or significant disability/incapacity" in the definition of "Serious" refers to the substantial disruption of a person's ability to conduct his/her normal life. Did the condition or event cause the participant to be permanently disabled, physically or mentally?
- **Requires in-patient hospitalization or prolongs existing hospitalization** Note: the term "hospitalization" in the definition of "Serious" refers to a condition or event that requires or prolongs inpatient hospitalization. It does not refer to a visit to the hospital emergency room. The participant must have been admitted to the hospital (usually

UNCONTROLLED COPY

includes an overnight stay). Any condition or event requiring a participant to be hospitalized qualifies as a Serious event, unless the participant is hospitalized for the following reasons:

- Surgery or procedure planned prior to the participant's entry into the trial, unless the timing of this intervention has been brought forward due a worsening of the original condition since the start of the trial.

- Elective (i.e., beneficial to the volunteer, but not essential for survival) treatment of an ongoing previous condition is NOT considered Serious. .

- Reasons other than an adverse event, for example, social factors such as a family can no longer look after a volunteer, it is too far to travel back and forth from home to the hospital for a series of treatments related to an ongoing previous condition, these should NOT be considered Serious Adverse Events

- **Is a congenital anomaly/birth defect** Note: the term "congenital anomaly/birth defect" in the definition of "Serious" refers to something different, abnormal, peculiar that exists at or dating from birth of a child born to the volunteer after vaccination.

- **Any other important medical condition** Note: Medical and scientific judgment should be exercised in deciding whether expedited reporting is appropriate in other situations, such as Important Medical Events that may not be immediately life-threatening or result in death or hospitalization, but may jeopardize the participant or may require intervention to prevent one of the other outcomes listed in the definition above. These events should be considered Serious and it should be documented why such an event was considered to be an SAE. Examples are allergic bronchospasm requiring intensive emergency treatment, anaphylaxis, seizures or blood dyscrasias, autoimmune disease that did not result in hospitalization or development of drug dependency.

10.4 Relationship to Investigational Product

The relationship of an (S) AE is assessed and determined by the principal investigator or designee. All medically indicated and available diagnostic methods (e.g. lab, blood smear, culture, X-ray, etc.) should be used to assess the nature and cause of the AE/SAE. Best clinical and scientific judgment should be used to assess relationship of AE/SAEs to the investigational vaccine and/or other cause.

The following should be considered for the assessment of relationship of adverse events to the investigational product:

- Presence/absence of a clear temporal (time) sequence between administration of the investigational vaccine and the onset of AE/SAE
- Presence/absence of another cause that could more likely explain the AE/SAE (concurrent disease, concomitant medication, environmental or toxic factors, etc.)
- Whether or not the AE/SAE follows a known response pattern associated with the vaccine

The relationship assessment should be reported as one of the following:

Not Related: clearly explained by another cause (concurrent disease, concomitant medication, environmental or toxic factors, etc.).

UNCONTROLLED COPY

Unlikely: more likely explained by another cause (concurrent disease, concomitant medication, environmental or toxic factors, etc.).
Possibly: equally likely explained by another cause but the possibility of the vaccine relationship cannot be ruled out (e.g. reasonably well temporally related and/or follows a known vaccine response pattern but equally well explained by another cause).
Probably: more likely explained by the vaccine (e.g. reasonably well temporally related and/or follows a known vaccine response pattern and less likely explained by another cause).
Definitely: clearly related and most likely explained by the vaccine

For the purpose of expedited safety reporting, all possibly, probably or definitely related SAEs are considered vaccine related SAEs.

10.5 Reporting Serious Adverse Events

Serious Adverse Events (see Section 10.3) should be recorded on the IAVI SAE Reporting Form and reported to IAVI within 24 hours of the trial site becoming aware of the event. SAEs should be entered in the database as soon as possible.

Notification must be made by fax followed by telephone contact.

The minimum information required in reporting a SAE are the volunteer identification number, date of birth, gender, event description (in as much detail as is known at the time), onset date of event (if available), reason event is classified as serious, date of last vaccination, reporting source (name of principal investigator or designee), causality in investigator's opinion and name of investigator.

Serious Adverse Event reporting numbers and contacts are

SAE Telephone Hotline: + 1 212 847 1110

Fax Number: + 1 888 317 4215

The IAVI SAE Reporting Form should be completed with all the available information at the time of reporting. The principal investigator or designee is required to write a detailed written report with follow up to resolution of the SAE (i.e., the volunteer recovers or dies, or the condition becomes chronic but relatively stable).

The principal investigator will notify the local IRB/IEC of SAEs according to their requirements. The sponsor will notify the Data and Safety Monitoring Board (DSMB) and the regulatory authorities.

When appropriate, the sponsor will also notify the regulatory authority and other trial site where the investigational product is being tested.

More details on SAE definitions and reporting requirements are provided in the SAE Reporting Guidelines (see Study Operations Manual).

UNCONTROLLED COPY

10.6 Clinical Management

Adverse events (AEs) will be managed by the clinical trial team who will assess and treat the volunteer as appropriate, including referral to an independent physician and/or health department. If any treatment/medical care is required as a result of the harm caused by the study vaccine or study procedures, this will be provided free of charge.

Study injections will be discontinued if the volunteer develops a disease, condition or adverse event, regardless of relationship to the vaccine, if in the opinion of the principal investigator or designee further injections will jeopardize the safety of the volunteer (see section 12.0 for details).

10.7 Pregnancy

If a female volunteer becomes pregnant during the study injections will be discontinued and the volunteer followed until the end of pregnancy or study completion, whichever occurs last. Pregnancy is not considered to be an adverse event. If a female volunteer becomes pregnant during the trial, it is the responsibility of the principal investigator or designee to report the pregnancy promptly to IAVI using the designated case report forms. However, serious complications of pregnancy that meet SAE criteria specified in the section 10.3 of this Protocol (e.g. eclampsia, spontaneous abortion, etc.) should also be reported as SAEs.

10.8 Evaluation for Intercurrent HIV Infection

HIV infection cannot be caused by the vaccine. If a volunteer is found to be HIV-infected through exposure in the community, study vaccinations must be discontinued and the volunteer followed according to procedures described in Section 11.3. Intercurrent HIV infection in study volunteers, although not considered an SAE, must be reported promptly to IAVI using the designated forms. However, serious medical conditions associated with the HIV infection that meet SAE criteria specified in the section 10.3 of this Protocol (e.g. sepsis, PCP pneumonia, etc.) should also be reported as SAEs using SAE Report From.

11.0 MANAGEMENT OF HIV ISSUES DURING AND FOLLOWING STUDY

11.1 HIV Testing

Volunteers will be tested for HIV-1 and HIV-2 antibodies as indicated in the Schedule of Procedures (Appendix A) or as needed, by an independent lab, if medical or social circumstances arise.

If the routine post-vaccination HIV Antibody test is positive, a predetermined algorithm will be followed to distinguish between an immune response to the Investigational Product from an HIV infection through exposure in the community.

To prevent unblinding of the volunteer and site personnel, an independent physician will review and interpret HIV test results in regards to a possible vaccine response. The results will be

UNCONTROLLED COPY

reported to the site as "HIV-infected" or "Not infected". If a volunteer is found to be HIV-infected, a newly drawn blood specimen will be collected for confirmation and the volunteer will be referred for appropriate medical care.

Volunteers who develop a vaccine-induced HIV positive result rather than a true HIV infection (through community exposure) will have their test result reported as “not infected” (to prevent unblinding of volunteer and staff) and will be followed up until the test becomes negative.

Should a volunteer require an HIV test outside the study for personal reasons, it is recommended that the volunteer contact the site staff first. The HIV test can be drawn at the clinical site and then processed at the independent laboratory as above. Written evidence of HIV status (HIV-infected or HIV-uninfected) will be provided upon request.

After the conclusion of the study, after un-blinding the results, all volunteers will be provided the written HIV-antibody response results.

11.2 Social Discrimination as a Result of an Antibody Response to Investigational Product

The aim is to minimize the possibility of social discrimination in volunteers who develop vaccine-induced HIV antibodies and test positive on a diagnostic HIV antibody test. Appropriate diagnostic HIV testing and certification will be provided both during and after the study as needed. In addition, a letter stating that the individual has participated in a vaccine trial, giving a contact number in case of medical emergency will be provided.

11.3 HIV Infection

Volunteers who are found to be HIV infected at screening and volunteers who acquire HIV infection during the study will be managed in the following way:

11.3.1 Counseling

The volunteer will be counseled by the study counselors. The counseling process will assist the volunteer with the following issues:

- Psychological and social implications of HIV infection
- Who to inform and what to say
- Implications for sexual partners
- Implications for child-bearing
- Avoidance of transmission to others in future

11.3.2 Referral for Support and/or Care

Volunteers will be referred to a patient support centre or institution of his/her choice for a full discussion of the clinical aspects of HIV infection. Referral will be made to a designated physician or centre for discussion of options of treatment of HIV-infection.

HIV-infected pregnant women will be referred for prenatal care and to a program for the Prevention of Mother to Child Transmission (PMTCT). The pregnant volunteer will be followed according to timeline as specified in Section 10.7.

UNCONTROLLED COPY

12.0 DISCONTINUATION OF VACCINATIONS AND/OR WITHDRAWAL FROM STUDY

12.1 Discontinuation of Further Vaccinations

Volunteers will be discontinued from further vaccination for any of the following reasons:

1. A disease or condition or an adverse event that may develop, regardless of relationship to the candidate vaccine, if the principal investigator or designee is of the opinion that further vaccination will jeopardize the safety of the volunteer.

2. An abnormal laboratory event, based on the following criteria, defined in Section 10.5:

   - For a mild laboratory abnormality, volunteers may be vaccinated only if the event is judged to be not clinically significant by the principal investigator or designee,

   - For a moderate laboratory abnormality, the laboratory test must be repeated and the event determined to be resolved in the opinion of the principal investigator or designee prior to vaccination,

   - For a severe laboratory abnormality, even if resolved, the IAVI Medical Monitor must be consulted before making a decision to vaccinate.

3. A severe local reactogenicity event involving the major circumference of the arm, not resolving within 72 hours.

4. Anaphylaxis; bronchospasm; laryngeal edema; collapse; convulsions or encephalopathy.

5. Life threatening medical event following vaccination unless not related to the investigational product.

6. Any immediate hypersensitivity reaction judged due to investigational product (e.g. anaphylaxis).

7. Pregnancy.

8. Intercurrent HIV Infection.

9. Volunteer request to discontinue further vaccination.

Any volunteer discontinuing further vaccination or being considered for discontinuation of vaccine will be discussed with the IAVI Medical Affairs Expert.

12.2. Follow up after discontinuation of further vaccinations

Any adverse event resulting in the discontinuation of a volunteer's vaccinations will be followed up until resolution or until the adverse event is judged by the principal investigator or designee to have stabilized where possible. Immunological monitoring will continue, provided the

UNCONTROLLED COPY

volunteer is willing. The frequency of assessments will be determined by the Trial Steering Committee.

A pregnant volunteer will be followed until the end of the study and until delivery, if delivery occurs after the study has ended. Approximately 2-4 weeks after delivery, the baby will be examined by a pediatrician to assess the health status of the baby. The health status of the baby will be reported to IAVI.

Follow-up of HIV-infected individuals who have received investigational product will be determined by the Trial Steering Committee.

The date and reason for discontinuation of vaccination should be collected.

12.3 Withdrawal from the Study (Early Termination)

Volunteers may be withdrawn from the study permanently for the following reasons:

1. If the volunteer wishes to do so, for any reason at any time.
2. Following an adverse event at the discretion of the investigator (or designee) discretion.
3. The principal investigator or designee has reason to believe that the individual is not complying with the protocol.

12.4 Follow-up after withdrawal from the study (Early Termination)

Any adverse event resulting in the withdrawal of a volunteer will be followed up until resolution or until the adverse event is judged by the principal investigator or designee to have stabilized where possible.

At the time of the withdrawal, provided the volunteer is willing, all the requested termination visit procedures will be performed according to the Schedule of Procedures (Appendix A).

The date and reason for withdrawal from the study (early termination) should be collected. Volunteers who are withdrawn from the study (early termination) will not be replaced, but, wherever possible, will be followed up to the time of their final planned visit.

13.0 DATA HANDLING

13.1 Data Collection and Record Keeping at the Study Site

Data Collection: All study data will be collected by the clinical study staff using designated source documents and entered onto the appropriate CRFs. CRFs will be provided by IAVI and should be handled in accordance with the instructions from IAVI. All study data must be verifiable to the source documentation. A file will be held for each volunteer at the clinic(s) containing all the source documents. Source documentation will be available for review to ensure that the collected data are consistent with the CRFs.

All source documents and other supporting documents will be kept in a secure location and separate from volunteer identification information (name, address, etc.) to ensure confidentiality.

UNCONTROLLED COPY

Standard GCP practices will be followed to ensure accurate, reliable and consistent data collection.

Source documents include but are not limited to:

- Signed Informed Consent Documents
- Dates of visits including dates of vaccinations
- Documentation of any existing conditions or past conditions relevant to eligibility
- Reported laboratory results
- All adverse events
- Concomitant medications
- Local and systemic reactogenicity

13.2 Data Collection and Transfer at the IAVI Core Laboratory

Data generated at the IAVI Core laboratory will be transferred directly to the Data Coordinating Centre (EMMES Corporation).

13.3 Data Entry at the Study Site

Data will be entered into the EMMES internet-based Data Entry System (IDES). Consistency checks and range checks will be performed by data entry and supervisory personnel. Queries raised by the monitor or by EMMES will be directed to the investigators and study staff.

13.4 Data Handling and Analysis

The statistician at the Data Coordinating Center (EMMES Corporation), in collaboration with the principal investigators (or designees) and the sponsor, will create tables according to a data analysis plan that has been reviewed and agreed to by the principal investigators (or designees) and IAVI. The EMMES Corporation will conduct the data analysis and will provide interim and final study reports for the DSMB, IAVI, principal investigators (or designees) and the regulatory authorities as appropriate. Prior to an analysis, additional monitoring visits will take place if necessary to validate the data held on the database, as well as all consent forms and dispensing records. Data files will be prepared by EMMES from a 'frozen' dataset for that particular analysis.

Unblinded data will be seen only by the DSMB prior to unblinding the trial. Preliminary grouped data analyses may be done without unblinding the volunteers, principal investigators or laboratory personnel to individual assignments.

14.0 STATISTICAL CONSIDERATIONS

14.1 Sample Size

The study uses a dose-escalation design of ADMVA vaccine candidate or placebo administered intramuscularly three times during the course of the study. The study investigates three dose levels of ADMVA vaccine, 1×10^7, 5×10^7 and 2.5×10^8 pfu. Each dose group consists of 16 volunteers randomized in a 3:1 ratio of active vaccine to placebo.

A total of up to 48 volunteers will be enrolled in the study; 36 volunteers will be given active vaccines and 12 volunteers will be given placebo. The small sample size is appropriate for an

UNCONTROLLED COPY

exploratory dose-escalation study of a novel product while safety and tolerability and immunogenicity of the vaccine are investigated.

14.2 Statistical Power and Analysis

Vaccine Safety and Tolerability:

The rate of local and systemic reactogenicity events will be used to assess the differences between dose groups. The sample size of the study was restricted by safety considerations. Nevertheless, the study has at least 80% power (at α=0.05 confidence level of an exact one-sided trend test) to detect the difference of 41 percentage points or more in the rate of events between the active (n_1=36) and the placebo (n_2=12) groups. The power of the statistical comparison was computed assuming 10% rate of events in the placebo group.

The rate of Serious Adverse Events related to vaccine will be used as one measure of the safety of the candidate vaccine. Adverse Events that may be temporarily incapacitating (for example, loss or cancellation of work or social activities), which could make a vaccine impractical for large scale use if they occur in more than a small proportion of cases, will also be assessed.

All adverse events will be reported, grouped as to whether or not they qualify as SAEs, their severity assessment, and their relationship to vaccine (as judged by the investigator and reviewed by the sponsor and the DSMB). The following example illustrates the limited statistical power of this initial Phase 1 study.

Prior to the enrollment of the medium dose level group:
If none of the volunteers receiving the vaccine experiences an SAE related to vaccine (n=12), the 95% upper confidence bound for the rate of these adverse events in the population is 0.22.

Prior to the enrollment of the highest dose level group:
If none of the volunteers receiving the vaccine experiences an SAE related to vaccine (n=24), the 95% upper confidence bound for the rate of these adverse events in the population is 0.12. Restricted to the medium dose level group (n=12), the upper confidence bound would be 0.22.

After completion of the study:
If none of the volunteers receiving the vaccine experiences an SAE related to vaccine (n=36), the 95% upper confidence bound for the rate of these adverse events in the population is 0.08. Restricted to the two highest dosage level groups (n=24), the upper confidence bound would be 0.12. Restricted to the highest dose level group (n=12), the upper confidence bound would be 0.22.

Vaccine Immunogenicity:

Cellular immune responses will be analyzed using binomial methods to examine for the presence or absence of HIV-specific T-cell responses quantified by ELISPOT and cytokine flow cytometry (CFC). Presence or absence of binding and neutralizing antibodies will be also analyzed. Assays will be performed in a similar fashion in all volunteers. Because of the small sample sizes and multiple epitopes, the results will be primarily descriptive. Nevertheless, the study has at least 80% power (at α=0.05 confidence level of an exact one-sided trend test) to detect the difference of 38 percentage points or more in the response rate between the active

UNCONTROLLED COPY

(n_1=36) and the placebo (n_2=12) groups. The power of the statistical comparison was computed assuming 5% response rate in the placebo group. The study has at least 80% power at the 5% (10%) alpha level, using an exact one-sided test, to detect a 47% (44%) or more difference in response rates between any two dose groups (n_1= n_2=12).

To test for a significant trend in the number of positive responses at the end of the study, there is at least 80% power at the 5% (10%) alpha level of detecting response rates of 0.100, 0.198, 0.355, 0.551 (0.100, 0.183, 0.311, 0.476), corresponding to a slope of 80% (70%). These calculations assume a 10% response rate in the placebo group and equally spaced scores of 1, 2, 3 & 4 assigned to the groups, with 1 assigned to placebo and 4 to the highest dose group.

Based on the previous experience with IAVI Phase I vaccine studies, it is expected that the amount of missing, unused or spurious data will be insignificant. Unused and spurious data will be listed separately and excluded from the statistical analysis. Missing data will be excluded from the statistical analysis. A data analysis plan will be developed and agreed upon by IAVI and the investigators prior to unblinding.

15.0 QUALITY CONTROL AND QUALITY ASSURANCE

To ensure the quality and reliability of the data gathered and the ethical conduct of this trial, standard operating procedures have been developed for all clinic and laboratory procedures. Regular monitoring will be performed according to ICH-GCP. as described in section 13.1. An independent audit of the study may be performed, if appropriate at the discretion of the sponsor.

The principal investigators, by signing the protocol, agree to facilitate study related monitoring, audits, IRB review and regulatory inspection(s) and direct access to source documents. Such information will be treated as strictly confidential and will under no circumstances be made publicly available.

16.0 DATA AND BIOLOGICAL MATERIAL

All data and biological material collected through the clinical trial shall be the joint property of the principal investigators or designees and IAVI.

The raw computerized data generated in this study will be held by EMMES Corporation on behalf of IAVI and the principal investigators or designees. The clinical sites will also hold the frozen data files and tables generated for the purposes of analysis. Principal investigators or designees will have access to the EMMES database with appropriate blinding.

17.0 ADMINISTRATIVE STRUCTURE

The principal investigator will be responsible for all aspects of the trial at the study site.

17.1 Trial Steering Committee (TSC)

The supervision of the trial and the operational activities will be the responsibility of a Trial Steering Committee (TSC) consisting of the principal investigators and designees, and sponsor representatives. The TSC will meet regularly with representatives from the clinical, laboratory and data management teams.

UNCONTROLLED COPY

17.2 Data and Safety Monitoring Board (DSMB)

The DSMB will consist of independent individuals who are not involved in the trial. No investigators responsible for the clinical care of trial volunteers or representative of IAVI may be a member of the DSMB. However, the DSMB may invite the principal investigators or designees and an IAVI representative to an open session of the meeting to provide information on study conduct, present data or to respond to questions at an open portion of the DSMB meeting.

The review of trial data by the DSMB will take place at least 14 days after the 12th volunteer in each dose group receives the second injection before proceeding to the next dose group. Additional meetings will take place if there are indications for an interim review, which may be unblinded at the discretion of the DSMB.

17.2.1 Content of interim review

The DSMB will be asked to review on an interim basis:

- All severe clinical adverse/reactogenicity events judged by the principal investigator or designee to be possibly, probably or definitely related to vaccine.
- All severe laboratory adverse events confirmed on retest and judged by the principal investigator or designee to be possibly, probably, or definitely related to vaccine.

17.2.2 Indications for discontinuation of vaccinations in all volunteers

If there is one SAE graded as severe and judged as possibly, probably, or definitely related to study vaccine by the principal investigator or designee, the trial will be suspended pending a review by at least one member of the DSMB which may be unblinded at its discretion. Following this review, the DSMB member(s) will make a recommendation to the principal investigators and IAVI regarding the continuation of the trial.

18.0 INDEMNITY

The Sponsor and Institution are responsible to have appropriate liability insurance. For research-related injuries and/or medical problems determined to result from receiving the Investigational Product, treatment including necessary emergency treatment and proper follow-up care will be made available to the volunteer free of charge at the expense of the Sponsor.

19.0 PUBLICATION

A primary manuscript describing safety and immune responses in this trial will be prepared promptly after the data analysis is available, based on the data compiled by the IAVI statistical centre. Authors will be representatives of trial site, the statistical centre, the laboratories and IAVI, subject to the generally accepted criteria of contributions to the design, work, analysis and writing of the study. Manuscripts will be reviewed by representatives of each participating group.

20.0 ETHICAL CONSIDERATIONS

This study will be conducted in compliance with the protocol, ICH/GCP and applicable regulatory requirements. It will be reviewed and approved by the respective national authorities.

UNCONTROLLED COPY

The trial will not be initiated before the protocol and informed consent and volunteer information form have been reviewed and received approval/favorable opinion from the local IRB. Should a protocol amendment be made that needs IRB approval, the changes in the protocol will not be instituted until the amendment and revised informed consent (if appropriate) have been reviewed and received approval/favorable opinion from the local IRB. A protocol amendment intended to eliminate an apparent immediate hazard to volunteers may be implemented immediately providing the appropriate regulatory authorities and IRB are notified as soon as possible and an approval is requested. Protocol amendments for logistical or administrative changes only may be implemented immediately; the IRB need only to be informed.

The constitution of the IRB must meet the requirements of the participating country. A list of the IRB members with names and qualifications will be requested. If such a list is unavailable, the principal investigators or designees must provide the name and address of the IRB along with the statement from the IRB that it is organized according to GCP and the applicable laws and requirements of the participating country.

UNCONTROLLED COPY

REFERENCES:

1. AIDS epidemic update December 2002. Joint UNAIDS/WHO, December 2002

2. Beyrer C, Razak MH, Lisam K, Chen J, Lui W, Yu XF. Overland heroin trafficking routes and HIV-1 spread in south and south-east Asia. *AIDS 2000* Jan 7;14(1):75-83

3. Yu XF, Liu W, Chen J, Kong W, Liu B, Yang J, Liang F, McCutchan F, Piyasirisilp S, Lai S. Rapid dissemination of a novel B/C recombinant HIV-1 among injection drug users in southern China. *AIDS 2001* Mar 9;15(4):523-5

4. Piyasirisilp S, McCutchan FE, Carr JK, Sanders-Buell E, Liu W, Chen J, Wagner R, Wolf H, Shao Y, Lai S, Beyrer C, Yu XF. A recent outbreak of human immunodeficiency virus type 1 infection in southern China was initiated by two highly homogeneous, geographically separated strains, circulating recombinant form AE and a novel BC recombinant. *J Virol 2000* Dec;74(23):11286-95

5. Barouch DH, Santra S, Kuroda MJ, Schmitz JE, Plishka R, Buckler-White A, et al. Reduction of simian-human immunodeficiency virus 89.6P viremia in rhesus monkeys by recombinant modified vaccinia virus Ankara vaccination. J Virol 2001; 75:5151-5158.

6. Seth A, Ourmanov I, Schmitz JE, Kuroda MJ, Lifton MA, Nickerson CE, et al. Immunization with a modified vaccinia virus expressing simian immunodeficiency virus (SIV) Gag-Pol primes for an anamnestic Gag-specific cytotoxic T-lymphocyte response and is associated with reduction of viremia after SIV challenge. J Virol 2000; 74:2502-2509.

7. Redfield RR, Wright DC, James WD, Jones TS, Brown C, Burke DS. Disseminated vaccinia in a military recruit with human immunodeficiency virus (HIV) disease. N Engl J Med 1987; 316:673-676.

8. Meyer H, Sutter G, Mayr A. Mapping of deletions in the genome of the highly attenuated vaccinia virus MVA and their influence on virulence. J Gen Virol 1991; 72 (Pt 5):1031-1038.

9. Hanke T, Blanchard TJ, Schneider J, Ogg GS, Tan R, Becker M, et al. Immunogenicities of intravenous and intramuscular administrations of modified vaccinia virus Ankara-based multi-CTL epitope vaccine for human immunodeficiency virus type 1 in mice. J Gen Virol 1998; 79 (Pt 1): 83-90.

10. Seth A, Ourmanov I, Kuroda MJ, Schmitz JE, Carroll MW, Wyatt LS, et al. Recombinant modified vaccinia virus Ankara-simian immunodeficiency virus gag pol elicits cytotoxic T lymphocytes in rhesus monkeys detected by a major histocompatibility complex class I/peptide tetramer. Proc Natl Acad Sci U S A 1998; 95:10112-10116.

11. Stittelaar KJ, Kuiken T, de Swart RL, van Amerongen G, Vos HW, Niesters HG, van Schalkwijk P, van der Kwast T, Wyatt LS, Moss B, Osterhaus AD (2001). Safety of modified vaccinia virus Ankara (MVA) in immune-suppressed macaques. Vaccine. Jun 14;19(27):3700-9.

UNCONTROLLED COPY

12. Altenburger W, Suter CP, Altenburger J. (1989). Partial deletion of the human host range gene in the attenuated vaccinia virus MVA. Arch Virol (1989);105(1-2):15-27

13. Mayr A, Stickl H, Muller HK, Danner K, Singer HM. The smallpox vaccination strain MVA: marker, genetic structure, experience gained with the parenteral vaccination and behavior in organisms with a debilitated defence mechanism. [Article in German] Zentralbl Bakteriol [B] 1978 Dec; 167(5-6): 375-90,

14. Cassimatis DC, Atwood JE, Engler RM, Linz P, Grabenstein JD, Vernalis MN. Smallpox vaccination and myopericarditis: a clinical review. J. Am. Coll. Cardiol. (2004); 43(9) 1503-1510.

15. Eckart RE, Lowe, SS, Atwood JE, Arness MK, Cassimatis DC, Campbell CL, Boyd SY, Murphy JG, Swerdlow DL, Collines LC, Riddle JR, Tornberg DN, Grabenstein JD, Engler RJM. Incidence and follow-up of inflammatory cardiac complication s after smallpox vaccination. J. Am. Coll. Cardiol. (2004); 44(1) 201-205.

16. Hanke T, Samuel RV, Blanchard TJ, Neumann VC, Allen TM, Boyson JE, et al. Effective induction of simian immunodeficiency virus-specific cytotoxic T lymphocytes in macaques by using a multiepitope gene and DNA prime-modified vaccinia virus Ankara boost vaccination regimen. J Virol 1999; 73:7524-7532.

17. Robinson HL, Montefiori DC, Johnson RP, Manson KH, Kalish ML, Lifson JD, et al. Neutralizing antibody-independent containment of immunodeficiency virus challenges by DNA priming and recombinant pox virus booster immunizations. Nat Med 1999; 5:526-534.

18. Hanke, T., Blanchard, T. J., Schneider, J., Ogg, G. S., Tan, R., Becker, M., Gilbert, S. C., Hill, A. V., Smith, G. L. & McMichael, A. (1998). Immunogenicities of intravenous and intramuscular administrations of modified vaccinia virus Ankara-based multi-CTL epitope vaccine for human immunodeficiency virus type 1 in mice. J Gen Virol 79, 83-90.

19. Seth A, Ourmanov I, Schmitz JE, Kuroda MJ, Lifton MA, Nickerson CE, et al. Immunization with a modified vaccinia virus expressing simian immunodeficiency virus (SIV) Gag-Pol primes for an anamnestic Gag-specific cytotoxic T-lymphocyte response and is associated with reduction of viremia after SIV challenge. J Virol 2000; 74:2502-2509.

20. Ourmanov, I., Bilska, M., Hirsch, V. M. & Montefiori, D. C. (2000). Recombinant modified vaccinia virus ankara expressing the surface gp120 of simian immunodeficiency virus (SIV) primes for a rapid neutralizing antibody response to SIV infection in macaques. J Virol 74, 2960-5.

21. Mayr A, Hochstein-Mintzel V, Stickl H. Abstammung, Eigenschaften und Verwendung des attenuierten Vaccinia-Stammes. Infection 1975; 3: 6-14

22. Mayr A, Danner K. Bedeutung von Tierpocken fur den Menschen nach Aufhebung der Pflichtimpfung gegen Pocken. Berl Muench Tieraerztl Wochenschr 1979; 92: 251-6.

23. Transgene Inc. Press release 2004

24. Corona Gutierrez CM, Tinoco A, Lopez Contreras M, Navarro T, Calzado P, Vargas L, Reyes L, Posternak R, Rosales R. Clinical protocol. A phase II study: efficacy of the

UNCONTROLLED COPY

gene therapy of the MVA E2 recombinant virus in the treatment of precancerous lesions (NIC I and NIC II) associated with infection of oncogenic human papillomavirus. Hum Gene Ther 2002; 13: 1127-40.

25. Moorthy VS, McConkey S, Roberts M, Gothard P, Arulanantham N, Degano P, Schneider J, Hannan C, Roy M, Gilbert SC, Peto TE, Hill AV. Safety of DNA and modified vaccinia virus Ankara vaccines against liver-stage P.falciparum malaria in non-immune volunteers. Vaccine. 2003 May 16; 21(17-18): 1995-2002.

26. McConkey SJ, Reece WH, Moorthy VS, Webster D, Dunachie S, Butcher G, Vuola JM, Blanchard TJ, Gothard P, Watkins K, Hannan CM, Everaere S, Brown K, Kester KE, Cummings J, Williams J, Heppner DG, Pathan A, Flanagan K, Arulanantham N,Roberts MT, Roy M, Smith GL, Schneider J, Peto T, Sinden RE, Gilbert SC, Hill AV. Enhanced T-cell immunogenicity of plasmid DNA vaccines boosted by recombinant modified vaccinia virus Ankara in humans. Nat Med. 2003 Jun; 9(6): 729-35. Epub 2003 May 25.

27. Moorthy VS, Pinder M, Reece WH, Watkins K, Atabani S, Hannan C, Bojang K, McAdam KP, Schneider J, Gilbert S, Hill AV. Safety and immunogenicity of DNA/modified vaccinia virus ankara malaria vaccination in African adults. J Infect Dis. 2003 Oct 15; 188(8): 1239-44. Epub 2003 Sep 30.

28. Hanke T, McMichael AJ. Design and construction of an experimental HIV-1 vaccine for a year-2000 clinical trial in Kenya. Nat Med. 2000 Sep;6(9):951-5

29. Mwau M, Cebere I, Sutton J, Chikoti P, Winstone N, Wee E.G-T, Beattie T, Chen Y-H, Dorrell L, McShane H, Schmidt C, Brooks M, Patel S, Roberts J, Conlon C, Rowland-Jones S, Bwayo J, McMichael A, Hanke T. A human immunodeficiency virus 1 (HIV-1) clade A vaccine in clinical trials: stimulation of HIV-specific T-cell responses by DNA and recombinant modified vaccinia virus Ankara (MVA) vaccines in humans. J.Gen.Virol. 2004: Vol. 85

30. Keefer MC, McElrath MJ, Weinhold K, Gorse GJ, Mulligan M, Francis D, Panicali D: A phase I trial of vaccinia-env/gag/pol (TCB-3B) given by alternative routes, boosted with rgp 120. Int Conf AIDS.1998;12: 278 (abstract number 496/28199).

31. Graham BS, Belshe RB, Clements ML, Dolin R, et al. Immunization of Vaccinia-Naïve Adults with Human Immunodeficiency Virus Type 1 gp160 Recombinant Vaccinia Virus in a Blinded, Controlled, Randomised Clinical Trial. J Inf Dis 1992;166:244-52.

32. McElrath, M. J., Corey, L., Berger, D., Hoffman, M. C., Klucking, S., Dragavon, J., Peterson, E. & Greenberg, P. D. (1994). Immune responses elicited by recombinant vaccinia-human immunodeficiency virus (HIV) envelope and HIV envelope protein: analysis of the durability of responses and effect of repeated boosting. J Infect Dis 169, 41-7.

33. Clements-Mann, M. L., K. Weinhold, et al. (1998). “Immune responses to human immunodeficiency virus (HIV) type 1 induced by canarypox expressing HIV-1MN gp120, HIV-1SF2 recombinant gp120, or both vaccines in seronegative adults. NIAID AIDS Vaccine Evaluation Group.” J Infect Dis 177(5): 1230-46.

34. Belshe, R. B., Gorse, G. J., Mulligan, M. J., Evans, T. G., Keefer, M. C., Excler, J. L., Duliege, A. M., Tartaglia, J., Cox, W. I., McNamara, J., Hwang, K. L., Bradney, A.,

UNCONTROLLED COPY

Montefiori, D. & Weinhold, K. J. (1998). Induction of immune responses to HIV-1 by canarypox virus (ALVAC) HIV-1 and gp120 SF-2 recombinant vaccines in uninfected volunteers. NIAID AIDS Vaccine Evaluation Group. Aids 12, 2407-15.

35. Belshe, R. B., Stevens, C., Gorse, G. J., Buchbinder, S., Weinhold, K., Sheppard, H., Stablein, D., Self, S., McNamara, J., Frey, S., Flores, J., Excler, J. L., Klein, M., Habib, R. E., Duliege, A. M., Harro, C., Corey, L., Keefer, M., Mulligan, M., Wright, P., Celum, C., Judson, F., Mayer, K., McKirnan, D., Marmor, M. & Woody, G. (2001). Safety and immunogenicity of a canarypox-vectored human immunodeficiency virus Type 1 vaccine with or without gp120: a phase 2 study in higher- and lower-risk volunteers. J Infect Dis 183, 1343-52.

36. Evans, T. G., Kallas, E. G., Campbell, M., Andrews, J., Schwartz, D., Keefer, M. & Caudrelier, P. (2001). Evaluation of canarypox-induced CD8(+) responses following immunization by measuring the effector population IFN-gamma production. Immunol Lett 77, 7-15.\

37. Evans, T. G., Keefer, M. C., Weinhold, K. J., Wolff, M., Montefiori, D., Gorse, G. J., Graham, B. S., McElrath, M. J., Clements-Mann, M. L., Mulligan, M. J., Fast, P., Walker, M. C., Excler, J. L., Duliege, A. M. & Tartaglia, J. (1999). A canarypox vaccine expressing multiple human immunodeficiency virus type 1 genes given alone or with rgp120 elicits broad and durable CD8+ cytotoxic T lymphocyte responses in seronegative volunteers. J Infect Dis 180, 290-8.

38. Salmon-Ceron D.,Excler, J. L., Finkielsztejn, L., Autran, B., Gluckman, J. C., Sicard, D., Matthews, T. J., Meignier, B., Valentin, C., El Habib, R., Blondeau, C., Raux, M., Moog, C., Tartaglia, J., Chong, P., Klein, M., Milcamps, B., Heshmati, F. & Plotkin, S. (1999). Safety and immunogenicity of a live recombinant canarypox virus expressing HIV type 1 gp120 MN MN tm/gag/protease LAI (ALVAC-HIV, vCP205) followed by a p24E-V3 MN synthetic peptide (CLTB-36) administered in healthy volunteers at low risk for HIV infection. AGIS Group and L'Agence Nationale de Recherches sur Le Sida. AIDS Res Hum Retroviruses 15, 633-45.

39. Excler JL, Plotkin S. The prime-boost concept applied to HIV preventive vaccines. AIDS 1997; 11 Suppl A:S127-S137

40. Mugerwa, R. D., P. Kaleebu, et al. (2002). “First trial of the HIV-1 vaccine in Africa: Ugandan experience.” BMJ 324(7331): 226-9.

APPENDIX A: SCHEDULE OF PROCEDURES

Visit Day (D)/Week (W)/Month (M)	D – 42 SCREEN	D0	D 3 PHONE	D 7	D 14	D28 (M1)	D 31 PHONE	D 35	D 42	D84 (M3)	D168 (M6)	D 171 (Phone)	D 182	D 196 (M7)	M 9	M 12	M 18 ET[a]
Visit Windows (Days)			± 1	± 2	± 2	± 3	± 1	± 2	± 2	± 3	± 7	± 1	± 2	± 3	± 7	± 7	± 7
Informed Consent	X																
ADMVA Vaccine/Placebo		X				X					X						
Medical History	X	X		X	X	X			X	X	X		X	X	X	X	X
General Physical Exam	X														X	X	X
Directed Physical Exam		X		X		X		X[d]	X	X	X		X				
Pregnancy Test (women)	X	X				X					X					X	X
Pregnancy Avoidance Counseling	X	X				X					X						
Risk Reduction and Safe Sex Counseling	X	X				X				X	X				X	X	X
HIV Testing (ref 11.1) Pre-/Post HIV test Counseling[b]	X	X				X				X	X				X	X	X
Adverse Events		X	X	X	X	X	X	X[d]	X	X	X	X	X	X	X		
Serious Adverse Events		X	X	X	X	X	X	X[d]	X	X	X	X	X	X	X	X	X
Vital Signs (pre and 30-45 minute post vaccination)		X				X					X						
Local and Systemic Reactogenicity Assessment (pre&post vaccination)		X	X	X	X	X	X	X[d]	X		X	X	X				
Hep BsAg, anti-HepC, syphilis	X																
HLA Typing**																	
Hematology and Clinical Chemistry	X	X		X		X			X	X	X		X		X	X	X
Immunology (CD4/CD8)		X									X						X
Antibodies to MVA vector		X				X				X	X				X		X
Urine dipstick	X	X		X		X			X		X		X		X	X	X
Cardiac Troponin I	X				X				X				X			X	X
ECG	X				X								X				X
PBMCs for Cellular immunogenicity assays (ELISPOT&CFC) and storage		X		X[c]	X			X[d]	X		X		X	X	X	X	X
Binding Antibody Responses		X			X					X	X		X	X	X	X	X
Neutralizing Antibody Responses		X			X					X	X		X	X	X	X	X
Serum, plasma storage		X			X					X	X		X	X	X	X	X

[a] ET = Early Termination [b] Post-test counseling will be offered after HIV test results are available, at the next study visit or when available according to site-specific SOPs.
** To be drawn once at any time point during study
X[c] for the volunteers in mid and high dosage groups only
X[d] for the volunteers in high dosage group only.

UNCONTROLLED COPY

APPENDIX B: ADVERSE EVENT SEVERITY ASSESSMENT TABLE

PARAMETER	MILD	MODERATE	SEVERE
**General Principle for Severity Assessment**	• Mild discomfort.<br>• Minimal or no limitation of daily activity.<br>• No need for medical intervention.	• Moderate discomfort.<br>• Some limitation of daily activities (e.g. able to work part-time)<br>• May require minimal or no medical intervention	▪ Severe discomfort.<br>▪ Marked limitation of daily activities (e.g. unable to work).<br>▪ Requires medical intervention.
**LOCAL REACTOGENICITY:**			
**Pain and tenderness**	Minimal discomfort associated with minimal or no limitation of use of arm	Moderate discomfort with some limitation of use of arm causing some interference with daily activities. May require single dose of analgesic or NSAID.	Severe discomfort with significant limitation of use of arm causing marked limitation of daily activities (e.g. unable to work). Requires medical intervention (e.g. repeated doses of analgesic or NSAID).
**Erythema (redness) or skin discoloration**	Light red blush up to 25% of the circumference of the upper arm	Marked redness involving up to 50% of the circumference of the arm	Brick red involving >50% of the circumference of the upper arm
**Edema (swelling)**	Light edema involving up to 25% of the circumference of the upper arm	Marked edema involving up to 50% of the circumference of the arm	Significant edema >50% of the circumference of the upper arm
**Induration**	Hardening under the skin <1.5 cm in diameter	Hardening under the skin 1.5-3.0 cm in diameter	Hardening under the skin >3.0 cm in diameter
**Skin damage (vesicle, ulcer)**	Vesicles or superficial disruption of epithelium < 1 cm	Vesicles or superficial disruption of epithelium 1-2 cm	Full thickness disruption of the epithelium (ulceration) > 2 cm
**Formation of crust, scab or scar**	Crust, scab or scar ≤ 2cm	Crust, scab or scar 2-4cm	Crust, scab or scar > 4cm

UNCONTROLLED COPY

SYSTEMIC REACTOGENICITY			
**Headache**	Minimal headache causing no interference with daily activities	Moderate headache causing some interference with daily activities. May require single dose of analgesic or NSAID.	Significant headache causing marked limitation of daily activities (e.g. unable to work). Requires medical intervention (e.g. repeated doses of analgesic or NSAID).
**Rigor (chills)**	Minimal discomfort causing no interference with daily activities	Moderate discomfort causing some interference with daily activities	Significant discomfort causing limitation with daily activities (e.g. unable to work).
**Malaise (generalized feeling of discomfort)**	Minimal discomfort causing no interference with daily activities	Moderate discomfort causing some interference with daily activities	Significant discomfort causing limitation with daily activities (e.g. unable to work).
**Fatigue (tiredness)**	Minimal discomfort causing no interference with daily activities	Moderate discomfort causing some interference with daily activities	Significant discomfort causing limitation with daily activities (e.g. unable to work).
**Myalgia (other than vaccination site)**	Minimal muscle pain causing no interference with daily activities	Moderate muscle pain causing some interference with daily activities. May require single dose of analgesic or NSAID.	Significant muscle pain causing marked limitation of daily activities (e.g. unable to work). Requires medical intervention (e.g. repeated doses of analgesic or NSAID).
**Arthralgia**	Minimal joint pain causing no interference with daily activities	Moderate joint pain causing some interference with daily activities. May require single dose of analgesic or NSAID.	Significant joint pain causing marked limitation of daily activities (e.g. unable to work). Requires medical intervention (e.g. repeated doses of analgesic or NSAID).
**Fever**	37.7–38.6°C (99.8–101.5°F)	38.7–39.3°C (101.6–102.7°F)	≥39.4°C (≥102.8°F)
**Nausea**	Minimal nausea causing no interference with daily activities	Moderate nausea causing some interference with daily activities...	Significant nausea causing marked limitation of daily activities (e.g. unable to work).

UNCONTROLLED COPY

**Vomiting**	Minimal nausea causing no interference with daily activities	Moderate nausea causing some interference with daily activities.	Significant nausea causing marked limitation of daily activities (e.g. unable to work). Requires medical intervention
**Pruritus**	Localized itching at vaccination site	Itching at the injected arm (beyond vaccination site)	Generalized itching
**Rash**	Localized maculopapular (non-urticarial) rash at the vaccination site area causing no interference with daily activities	Diffuse maculopapular (non-urticarial) rash at the injected arm (beyond vaccination site) causing some limitation of daily activities	Systemic maculopapular (non-urticarial) rash causing marked limitation of daily activities. Requires medical intervention
**Allergic reaction**	Localized urticaria at vaccination site causing no interference with daily activities	Diffuse urticaria at the injected arm (beyond vaccination site) and causing some limitation of daily activities	Systemic urticaria or angioedema causing marked limitation of daily activities. Requires medical intervention
**HEMATOLOGY**			
Hemoglobin	10.0 g/dL – 11.0 g/dL<br>OR<br>any decrease ≥ 2.5 g/dL	9.0 g/dL – 9.9 g/dL<br>OR<br>any decrease ≥ 3.5 g/dL	= or <8.9 g/dL<br>OR<br>any decrease ≥ 4.5 g/dL
WBC—Elevated	13,000–14,999/mm^3	15,000–19,999/mm^3	= or >20,000/mm^3
WBC—Decreased	2000–2499/mm^3	1500–1999/mm^3	= or <1499/mm^3
Absolute Neutrophil Count	1000–1300/mm^3	750–999/mm^3	= or <749/mm^3
Absolute Lymphocyte Count	600–649/mm^3	500–599/mm^3	= or <499/mm^3
Absolute CD4 Count (HIV Negative)	300–400/mm^3	200–299/mm^3	= or <199/mm^3
Platelets—Decreased	100,000–124,999/mm^3	50,000–99,999/mm^3	= or <49,999/mm^3
Platelets—Elevated	NA	550,000–600,000/mm^3	> 600,000/mm^3
**CHEMISTRY**			
BILIRUBIN (total)	1.0–1.5 x ULN	>1.5–2.5 x ULN	>2.5 x ULN
CREATININE	1.1–1.3 x ULN	>1.3–1.8 x ULN	>1.8 x ULN
AST (SGOT)	1.51–3.0 x ULN	>3.0–6.0 x ULN	>6.0 x ULN
ALT (SGPT)	1.51–3.0 x ULN	>3.0–6.0 x ULN	>6.0 x ULN

UNCONTROLLED COPY

**URINALYSIS**			
PROTEINURIA	1+ (30 mg/dl)	2–3+ (100-500mg/dl)	4+ (> 500mg/dl)
Random urine<br>24-hour urine	200 mg–500 mg loss/day	> 500 mg–1.0 g loss/day	> 1.0g loss/day
HEMATURIA (in the absence of vaginal bleeding)<br>By microscopic exam only	1+ (ca. 5–10 Ery/ml)<br>6-10 Ery/hpf	2+ (ca. 10–25 Ery/µl)<br>>10 Ery/hpf	3+ (ca. 50 Ery/µl)<br>Gross, with or without clots or RBC casts
GLUCOSURIA	1+ (30mg/dl, 2.8 mmol/l)	2+ (100mg/dl, 5.5mmol/l)	3+ (300mg/dl, 17 mmol/l)
LEUCOCYTURIA	1+ (ca. 10–25 Leuco/ml)	2+ (ca. 75 Leuco/ml)	3+ (ca. 500/ml)
**CARDIOVASCULAR**			
Hypotension	Transient orthostatic hypotension with heart rate increased > 20 beats/min **OR** Decreased by >10mm Hg systolic BP, No Rx required	Symptoms **OR** BP decreased by >20 mm Hg systolic, correctable with oral fluid Rx	IV fluid required **OR** Hospitalization
Hypertension	Asymptomatic, transient increase by >20 mmHg diastolic BP **OR** >150/100 if previously normal.<br>No treatment required.	Recurrent or persistent or symptomatic increase by >20 mmHg diastolic BP **OR** >150/100 if previously normal.<br>Treatment required.	More intensive treatment required **OR** more than one drug required **OR** requires acute treatment
Cardiac Arrhythmia	Asymptomatic with transient dysrhythmia causing no interference with daily activities.<br>No treatment required.	Notable symptoms causing some interference with daily activities.<br>Non-urgent treatment required	Symptomatic and incompletely controlled by medical or invasive treatment.
Pericarditis	Minimal asymptomatic effusion	More than minimal asymptomatic effusion requiring no treatment	Symptomatic effusion, pain, EKG changes.
Hemorrhage, blood loss	Asymptomatic and requiring no therapy	Mildly symptomatic	Gross blood loss **AND/OR** 1–2 units transfused

**GASTROINTESTINAL**			
Constipation	Minimally symptomatic. No medical intervention required.	Significant abdominal pain with impaction requiring prescription	Requiring disimpaction<br>**AND/OR**<br>Hospital treatment
Diarrhea	Mild or transient or intermittent episodes (2-3/day)of unformed stools resulting in minimal or no interference with daily activities	Persistent episodes (5-10/day)of unformed-to-watery stools resulting in greater than minimal interference with daily activities	Bloody diarrhea or >10/day episodes.<br>**AND/OR**<br>Orthostatic hypotension<br>**AND/OR**<br>Electrolyte imbalance requiring IV fluid/therapy
Oral Discomfort/Dysphagia	Mild discomfort, no difficulty swallowing	Difficulty swallowing but able to eat and drink	Unable to swallow solids
**NEUROLOGICAL**			
Neuropsychiatric/mood	Mild mood alteration not interfering with daily activities. No intervention required.	Depression or anxiety symptoms causing individual to seek attention and be treated with counseling and/or pharmacotherapy	Severe mood changes requiring additional medical intervention<br>**AND/OR**<br>Suicidal ideation/gesture
Paresthesia (burning, tingling, etc.)	Minimal discomfort resulting in minimal or no interference with daily activities	Notable symptoms resulting in greater than minimal changes in daily activities	Marked and persistent discomfort resulting in significant incapacity<br>**AND/OR**<br>Narcotic analgesia required for symptomatic improvement
Neuro-motor	Mild weakness resulting in minimal or no interference with daily activities	Moderate weakness resulting in greater than minimal interference with daily activities	Significant incapacity

UNCONTROLLED COPY

Neuro-sensory	Mild impairment (decreased sensation) resulting in minimal or no interference with daily activities	Moderate impairment resulting in greater than minimal interference with daily activities	Significant incapacity
Neuro-cerebellar	Slight incoordination **OR** Dysdiadochokinesia	Intention tremor **OR** Slurred speech **OR** Nystagmus	Ataxia requiring assistance to walk or arm incoordination interfering with daily life activities
**RESPIRATORY**			
Cough	Transient cough resulting in minimal or no interference with daily activities	Recurrent or persistent cough resulting in greater than minimal interference with daily activities	Uncontrolled cough causing significant interference with daily activities
Bronchospasm Acute	Transient not requiring treatment. FEV1 or peak flow reduced to 70–80%	Treatment required - normalizes with bronchodilator. FEV1 or peak flow 50–69%	More intensive treatment required - no normalization with bronchodilator. FEV1 or peak flow < 50%
Dyspnea	Dyspnea on exertion (such as using stairs)	Dyspnea with normal activity (such as walking)	Dyspnea at rest
**MISCELLANEOUS**			
Arthritis	Mild pain with no joint swelling. No interference with daily activities.	Moderate pain with inflammation, erythema, or joint swelling. Some interference with daily activities.	Severe pain with inflammation, erythema, or joint swelling causing significant incapacity.
Eye	Symptoms resulting in minimal or no interference with daily life activities	Notable symptoms resulting in greater than minimal interference with daily life activities	Symptoms (such as loss of vision, clinically diagnosed uveitis, or glaucoma) resulting in significant incapacity
Skin (general)	Localized, asymptomatic.	Diffuse with notable symptoms and/or some interference with daily activities.	Generalized, marked symptoms and/or significantly interference with daily activities.
**ADDITIONAL LABORATORY ABNORMALITIES**			
Fibrinogen—Elevated	450–600 mg/dL	601–650 mg/dL	> 650 mg/dL
Fibrinogen—Decreased	100–200 mg/dL	< 100 mg/dL	< 75 mg/dL

UNCONTROLLED COPY

Prothrombin Time (PT)	1.1–1.24 x ULN	1.25–1.49 x ULN	= or >1.5 x ULN
PTT	1.1–1.66 x ULN	1.67–2.33 x ULN	= or >2.34x ULN
BUN	25–30 mg/dL	31–40 mg/dL	= or >41 mg/dL
LDH	1.5–2.5 x ULN	2.6–3.5 x ULN	= or >3.6 x ULN
Hyponatremia	130–135 meq/L	123–129 meq/L	= or <124 meq/L
Hypernatremia	146–150 meq/L	151–157 meq/L	= or >158 meq/L
Hyperkalemia	5.0–5.5 meq/L	5.6–6.0 meq/L	= or >6.1 meq/L
Hypokalemia	3.2–3.4 meq/L	3.0–3.1 meq/L	= or <2.9 meq/L
PHOSPHATE Hypophosphatemia	2.0–2.4 mg/dL	1.5 –1.9 mg/dL	= or <1.4 mg/dL
Hypocalcemia	7.8–8.4 mg/dL	7.0–7.7 mg/dL	= or <6.9 mg/dL
Hypercalcemia	10.6–11.5 mg/dL	11.6–12.5 mg/dL	= or >12.6 mg/dL
Hypomagnesaemia	1.2 –1.4 meq/L	0.9–1.1 meq/L	= or <0.8 meq/L
Hypoglycaemia	3.1–3.6 mmol/l (55–64 mg/dL)	2.2–3.0 mmol/l (40–54 mg/dL)	= or <2.1 mmol/l (= or <39 mg/dL)
Hyperglycaemia (nonfasting and no prior diabetes	6.5–9.0 mmol/l (116–160 mg/dL)	9.1–14.0 mmol/l (161–250 mg/dL)	= or >14.1 mmol/l (= or >251 mg/dL)
TRIGLYCERIDES	NA	400–750 mg/dL	= or >751 mg/dL
Hyperuricemia	7.5–10.0 mg/dL	10.1–12.0 mg/dL	= or >12.1 mg/dL
GGT	1.25–2.5 x ULN	2.6–5.0 x ULN	= or >5.1 x ULN
Alkaline Phosphate	1.25–2.5 x ULN	2.6–5.0 x ULN	= or >5.1 x ULN
Amylase	1.1–1.5 x ULN	1.6–2.0 x ULN	= or >2.1 x ULN
Pancreatic amylase	1.1–1.5 x ULN	1.6–2.0 x ULN	= or >2.1 x ULN
Lipase	1.1–1.5 x ULN	1.6–2.0 x ULN	= or >2.1 x ULN

UNCONTROLLED COPY

APPENDIX C: SAMPLE INFORMED CONSENT FORM

This appendix to be made site-specific and attached to this protocol by site

Consent Information Sheet for Screening and Participation in a Study with an Experimental HIV Vaccine

Protocol: IAVI C002

Title: A Randomized, Placebo-Controlled, Dose-Escalating, Double-Blinded Phase 1 Study to Evaluate the Safety and Immunogenicity of a Modified Vaccinia Ankara (MVA) expressing HIV-1 Clade C env/gag-pol and nef-tat fusion genes (ADMVA) Vaccine Administered Intramuscularly to HIV-Uninfected, Healthy Volunteers.

You have been asked to participate in the investigational research study named above. You are being asked to join this research study voluntarily as a healthy individual, not as an individual at high risk of developing human immunodeficiency virus (HIV).

You have the right to know about the procedures, risks, hazards, discomforts, and possible benefits of this study to help you make an informed decision about whether or not you will participate in the study. Please read the information below and ask questions about anything you don't understand before deciding whether or not to take part. This informed consent form does not replace any other informed consent forms you have signed.

Purpose of this study
Over 40 million people worldwide are currently infected with HIV, the virus that causes AIDS (Acquired Immune Deficiency Syndrome). The number of new cases continues to rise at an alarming rate. Other infectious diseases, such as smallpox or poliomyelitis, have been controlled, or even eliminated, by vaccination programs. Many experts believe that an HIV vaccine offers the best hope for controlling the epidemic.

Many different possible HIV vaccines are currently being developed and tested. This research study is a phase one study (first in humans) to determine whether this candidate (experimental) HIV vaccine is safe for use. The study will also look at how your immune system responds to the vaccine. This trial is one stage of testing this vaccine in humans; further studies will have to be carried out to determine whether this vaccine protects the recipient from getting HIV or AIDS before the vaccine can be used in the general population.

Background
The vaccine was designed by scientists at the Aaron Diamond AIDS Research Center (ADARC) and was manufactured at Impfstoffwerk Dessau-Tornau GmbH (IDT). The study sponsor is the International AIDS Vaccine Initiative (IAVI), an international, scientific, non-profit organization, whose mission it is to develop a safe and effective preventive vaccine against HIV, the virus that causes AIDS. This project is one of several vaccine development programs of IAVI.

Selection of study participants
This study will enroll approximately 48 healthy HIV-1 and HIV-2 negative male or female volunteers at lower risk of HIV infection, aged between 18 and 40 years, who are well informed about the study and provide written informed consent.

Duration of the study
The entire study will last approximately 19 months, including screening.

Study procedures
Pre-screening
- Before screening, you will have the opportunity to obtain basic information about the details of the study. You will have the opportunity to talk to the study doctors and nurses and ask them questions.

Screening
- Screening will determine whether or not you are eligible for the study.
- If you agree to be screened, you will sign 2 copies of the Informed Consent Form confirming that you have been informed about the study and voluntarily agree to take part. One copy is for you to keep and one will be kept in your confidential study file. If you do not wish to keep your copy, you will sign a form that states you have declined to take your copy and we will keep it for you.
- You will be asked questions about your general health and your sexual behavior.
- Pre-HIV test counseling will be provided.
- Up to 28 mL (about 2 tablespoons) of blood will be drawn to test for HIV and other health conditions.
- An electrocardiogram (ECG) will be done to record your heart's electrical activity and rhythm. For the ECG, leads (suction cups, or stickers) will be placed on your chest, arms and legs and you will need to lie still for several seconds.
- A urine specimen will be collected for testing, including a pregnancy test for females with childbearing potential.
- The screening visit will take 1-2 hours.

Your participation at screening is entirely voluntary. Attending information seminars and undergoing prescreening or screening tests do not obligate you to continue participation in this vaccine study.

Study Participation
If you are found eligible through the screening process, you have the option of participating in the study.
- You will have another opportunity to talk to and ask questions of the study doctors and nurses. The Informed Consent Information Sheet will be reviewed with you again to ensure that you have been fully informed about the study.
- You need to know that:
    - Your participation is voluntary, that is, it is entirely up to you whether you choose to participate in this study or not.
    - Attending information seminars and undergoing prescreening or screening tests do not obligate you to join the vaccine study.
    - If you decide not to participate, none of your legal rights will be compromised.
    - You may withdraw your consent to participate at any time, for any reason, without prejudice.

UNCONTROLLED COPY

- If you decide not to participate in this research study, there might be another HIV vaccine trial in the future. Currently, there is no licensed HIV vaccine available.

- If you decide to participate in the study:
  - You will be assigned a study identifier.
  - On the day of the study vaccination visit you will need to be at the clinic for approximately 1-1/2 hours. A medical history, physical exam and blood and urine will be collected for testing prior to giving you the study vaccine.
  - You will receive three vaccinations given in alternating upper arms. The vaccinations will be given at the initial study visit following the screening visit, and then 4 weeks and 6 months later.
  - In addition to the vaccination visits, you will have 3 phone calls (2-4 days after each vaccination) and 10 scheduled visits to the clinic for medical evaluations spread over a period of 78 weeks (18 months). However, if you are enrolled in high dosage group, you will have one extra visit at day 7 after second vaccination. These follow-up visits will last about 30 minutes each.
  - If you are enrolled in the low dosage group, approximately 180 ml (about 12 tablespoons) of blood will be taken at the first vaccination visit. Approximately 120 ml (about 8 tablespoons) will be taken at 4 of your study visits. Approximately 110 ml (less than 8 tablespoons) will be taken at 4 of your study visits. Approximately 20 ml (less than 2 tablespoons) of blood will be taken at 2 of your study visits. Approximately 10 ml (less than 1 tablespoon) will be taken at one of your study visits. If you are enrolled in mid dosage and high dosage group the blood volume to be collected at day 7 post 1st vaccination visit and day 7 post 2nd vaccination (high dose group) might be increased compared to the volume collected for low dosage group for the same time point. However, total blood volume to be collected for combined day 7 and day 14 post 1st and 2nd vaccination in mid and high dosage groups, will be adjusted to be equal to the total blood volume drawn at Day 14 post vaccination time points in low dosage group. Hence the total blood volume to be collected in 8 week period for all dosage groups will be the same.
  - An ECG test will be done 2 weeks following the 1st and 3rd vaccinations and at the end of the study.
  - Females of childbearing potential will have a urine pregnancy test prior to receiving each vaccine dose. This test is being done because the safety of the vaccine for pregnant women and their fetuses is not yet known. There will also be pregnancy tests at the Month 12 and Month 18 study visits. If you become pregnant during the study, you will not receive further vaccinations if you have not yet completed the vaccine schedule, and you will be followed up until the delivery of the baby, and the baby will be examined by a pediatrician approximately 2-4 weeks after birth.

Study Vaccine

The vaccine being tested in this study is artificially made. This study will be the first time that human beings will be injected with this vaccine. It does not contain any material from live HIV. It does not contain blood or blood products, or material from individuals who are infected with HIV and does not contain material from individuals who have been found to be resistant to HIV. The vaccine contains a weakened live virus called Modified Vaccinia Ankara or MVA. This MVA is a modified form of the original MVA vaccine, which was given to 120,000 humans and has been shown to be safe as part of the global smallpox eradication program in the 1970s. Individuals who have previously received smallpox vaccination may participate in the study. This vaccine (ADMVA) has not been tested in humans before. In each dose group, 4 of the 16

UNCONTROLLED COPY

subjects will receive placebo and not the study vaccine. The placebo is an inactive salt-water solution that looks like the active study vaccine.

This research study involves an experimental vaccine, called "ADMVA". The word "experimental" is used when the United States Food and Drug Administration has not approved a drug for use in the general public. However, we have told the United States Food and Drug Administration about this research study, and it has allowed this vaccine to be used in the study.

It is absolutely NOT POSSIBLE to get HIV infection from this vaccine.

Study Design
This study is called a dose-escalation study. The dose of the vaccine will be incrementally increased after the data collected on the volunteers is reviewed to ensure that it is safe to continue with the study.

Volunteers will be screened for the study on a first-come, first-served basis. If you qualify for the study and wish to join, you will then be assigned randomly by a computer (which means by chance or like in a lottery) to receive either study vaccine or placebo. There is a four out of five chance that you will receive the study vaccine and a one out of five chance that you will receive the placebo. A placebo looks like the vaccine, but it does not contain the vaccine. All volunteers will receive three injections. There will be 3 groups in the study. Each group will have 16 volunteers.

- Group 1: 12 volunteers will receive the lowest dose of the vaccine. 4 volunteers will receive the placebo.
- Group 2: 12 volunteers will receive the middle dose of the vaccine. 4 volunteers will receive the placebo.
- Group 3: 12 volunteers will receive the highest dose of the vaccine. 4 volunteers will receive the placebo.

The information as to which group (vaccine or placebo) you are assigned to (the code) is kept secret in a sealed envelope. Only in the case of a serious medical event may the code be broken by a principal investigator after consultation and agreement with other physicians and scientists on the study team.

The investigators and volunteers will not know whether a volunteer has received vaccine or placebo until the end of the study. Only the people who generate the code list, the people who package and label the vaccines and possibly the Data and Safety Monitoring Board will know the code. None of these people will be involved in any clinical or laboratory evaluations during the trial.

Storage of Blood
After completion of the initial laboratory tests on your blood specimens, some of the remaining blood will be stored. A unique study identifier rather than your name will be used to label this blood. The stored samples may be used in the future for follow-up safety testing, quality control purposes and other tests related to approved HIV vaccine research and development, and may therefore be shipped to independent national or international laboratories.

UNCONTROLLED COPY

Genetic Testing
A test will be conducted to determine your HLA type. HLA is a specific characteristic on the surface of your body cells. This information will help the researchers to understand differences in immune responses to the vaccine. The result of this test will be kept strictly confidential.

Risks and Discomforts
Blood tests for screening and study visits will be done by inserting a needle into your vein and can cause temporary local pain, bruising, and, rarely, infection. Following administration of the vaccine, you may experience pain, soreness, redness/discoloration and bruising around the site of the injection. With any new medicine or vaccine, there is a possibility of totally unexpected side effects, although previous testing indicates that this is unlikely.

This is the first study to test this vaccine in people. However, this vaccine has been tested in animals and was shown to be well tolerated. The local tolerability of ADMVA vaccine was tested in rabbits using the highest human clinical dose ever used. Rabbits were administered with ADMVA intramuscularly 3 times at 3 week intervals. ADMVA-induced microscopic changes observed at the injection sites were inflammatory and degenerative in nature. However, there were no clinically significant findings of either erythema or edema associated with histologic changes at the site of injection. By Day 46 the microscopic inflammatory changes localized to injection site had fully resolved at the site of Day 1 injection. People have received similar HIV vaccines made from MVA, and they were well-tolerated in the people who received them.

The MVA vaccine in this study is related to the small pox vaccine. Scientists recently learned that the vaccination that was used worldwide to protect against smallpox (vaccinia) might have caused heart problems in some of the people who received the vaccine. Although some people might not feel any problems, there may be changes on their ECG or blood tests. Although the experimental MVA vaccine used in this study was changed to prevent side effects caused by the smallpox vaccine, we do not know if it could cause any possible risk of heart problems. So far, no heart problems related to vaccine have been reported in the approximately 200 people who have received experimental MVA vaccines. However, people with current heart problems, or people who we think might be at risk for heart problems, are not allowed to join this study. In addition, people who join this study will be asked by clinic staff at every visit about symptoms that could be early signs of heart problems and we will watch carefully for heart problems.

This study focuses primarily on how well the vaccine is tolerated (vaccine safety) and is not looking to see if the vaccine can prevent HIV infection or disease. Until larger studies have been performed, we will not know whether this vaccine is effective in protecting you from getting HIV or AIDS or slowing HIV disease. Therefore you should continue to avoid any behavior that may put you at risk of contracting HIV. **YOU SHOULD NOT CONSIDER YOURSELF PROTECTED FROM HIV AFTER PARTICIPATING IN THIS STUDY.**

We do not know what effect the vaccine would have on an unborn child if given to a pregnant woman. Females of child-bearing potential should use a reliable form of contraception, as discussed with a nurse counselor, until 4 months after the last vaccination. A pregnancy test will be carried out at screening, prior to the vaccinations, and at 12 and 18 months after the first vaccination. Males who are not anatomically sterile should use condoms for 4 months after receiving the study vaccinations to avoid pregnancy in a spouse or partner.

Following vaccination you may test HIV positive on a routine HIV test. This does not mean that you have HIV or AIDS. It could mean that your body has been exposed to the vaccine and has produced antibodies to it. In case of a positive HIV test result, an independent laboratory will

UNCONTROLLED COPY

confirm whether you test positive as a result of the vaccination or whether you became infected with HIV through exposure in the community.

Should you get naturally infected with HIV due to exposure in the community; you will be referred for support and care that is standard.

Should you require an HIV test outside the study for whatever reason, we strongly recommend you contact the study team first. We will offer HIV testing at an independent laboratory that can distinguish between a positive result from a vaccine and a true HIV infection. To help avoid any problems, you will be offered a letter or identification card that shows that you have joined this study. A contact number in case of queries or medical emergencies will be provided.

It is unknown whether receiving this HIV vaccine will alter your immune response to any future HIV vaccine that you might receive.

You should not donate blood while you are participating in the study.

Benefits
There are no direct benefits for you in taking part in the study except that you will get information about your general health and your HIV status, and you will receive HIV counseling. However, the information that we gain from this study may help to develop an effective HIV vaccine, which would benefit others.

Injuries
We do not expect you to suffer any injury as a result of participating in this study. However, in case you are injured as a result of being in this study, you will be given the necessary treatment for your injuries without charge. This treatment will include any necessary emergency treatment and appropriate referral, as needed. If you have any symptoms or medical problems that you think are due to getting this vaccine, you should report them right away to ______________________ at telephone number ______________.

Circumstances for discontinuation or withdrawal from the trial
Your participation in the trial is completely voluntary. You can withdraw from the study at any time without giving a reason. Withdrawal will NOT compromise any rights you may have or influence any current or future medical care you may need.

You may be removed from the study without your consent for the following reasons:

- If your doctor feels that staying in the study is harmful to your health
- If you don't keep appointments
- If you have serious side effects from the vaccination
- If the Data and Safety Monitoring Board feels that the study should be stopped
- If the study sponsor decides to stop or cancel the study for any reason

New information
You will be told about any new information gained during the course of the study that might cause you to change your mind about staying in the study. You will be told about findings in this trial, and trials elsewhere, regarding the safety or effectiveness of this vaccine, as well as other HIV vaccines. You will also be told if a safe and effective HIV vaccine becomes available.

Supervision of the study
The trial will be supervised by a Trial Steering Committee. All data collected will be regularly checked by independent monitors and an independent Data and Safety Monitoring Board.

UNCONTROLLED COPY

Confidentiality
Your participation in the study will be kept strictly confidential. You will be identified by a unique identity number known only to you and the clinic staff. All information collected about you, as well as all results of laboratory tests will be marked by this number and kept strictly confidential. In addition to the study staff that you meet, there may be other staff from national or international government regulatory agencies, members of the Data and Safety Monitoring Board, monitors, auditors, inspectors, and representatives of the sponsor who will check the records to make sure that the trial was conducted properly. They are equally bound to respect your confidentiality. Your identity will not be disclosed in any publication or presentation of this study.

End of Study
An analysis of the results will be performed after all volunteers have completed the study. This analysis will take about 6 to 12 months to complete. You will be told whether you received the study vaccine or the placebo.

Reimbursement
You will be reimbursed $____ for the costs of travel, time, and childcare for each study visit.

Contact numbers
If you have any further concerns or questions about the study or during the study, please feel free to call and discuss them with us.

If you have any questions regarding the study or your participation in the study, you may call____.

If you would like to discuss your rights as a research subject or aspects concerning your participating in the study, you may contact ________________________________.

UNCONTROLLED COPY

INFORMED CONSENT FORM

IAVI C002

I, (name of subject) ....

of (address) ....

agree to take part in the research project entitled:

A Randomized, Placebo-Controlled, Dose-Escalating, Double-Blinded Phase 1 Study to Evaluate the Safety and Immunogenicity of a Modified Vaccinia Ankara (MVA) expressing HIV-1 Clade C env/gag-pol and nef-tat fusion genes (ADMVA) Vaccine Administered Intramuscularly to HIV-Uninfected, Healthy Volunteers.

I confirm that the nature and demands of the research have been explained to me and I understand and accept them. I understand that my consent is entirely voluntary and that I may withdraw from the research project if I find that I am unable to continue for any reason and this will not affect the legal rights I may otherwise have.

Participant:
Print Name: .... Signature: ....

Date: |__|__|/|__|__|__|/|__|__|__|__|Time: |__|__|: |__|__| (24 hours)
D D M M M/ Y Y Y Y

Person obtaining consent:
I have explained the nature, demands and foreseeable risks of the above research to the subject:

Print Name: .... Signature ....

Date: |__|__|/|__|__|__|/|__|__|__|__|Time: |__|__|: |__|__| (24 hours)
D D M M M/ Y Y Y Y

Principal Investigator or designee:

Print Name:.......................... Signature: ....

Date: |__|__|/|__|__|__|/|__|__|__|__|Time: |__|__|: |__|__| (24 hours)
D D M M M/ Y Y Y Y

Consent Copies

_____☐ I have been provided a copy of this signed and dated consent.
initials

_____☐ I have declined my copy of this signed and dated consent.
initials

UNCONTROLLED COPY
